# LncRNA SNHG1 contributes to sorafenib resistance by activating the Akt pathway and is positively regulated by miR-21 in hepatocellular carcinoma cells

**DOI:** 10.1186/s13046-019-1177-0

**Published:** 2019-05-03

**Authors:** Weidong Li, Xuesong Dong, Changjun He, Gang Tan, Ziyi Li, Bo Zhai, Jing Feng, Xian Jiang, Chang Liu, Hongchi Jiang, Xueying Sun

**Affiliations:** 1 The Hepatosplenic Surgery Center, The First Affiliated Hospital of Harbin Medical University, Harbin, 150001 China; 2 Department of General Surgery, The Fourth Affiliated Hospital of Harbin Medical University, Harbin, 150001 China; 3 Department of Surgery, The Third Affiliated Hospital of Harbin Medical University, Harbin, China

**Keywords:** Long non-coding RNA, Small nucleolar RNA host gene 1, MicroR-21, Akt, Hepatocellular carcinoma, Sorafenib, Drug resistance, Cellular signaling

## Abstract

**Background:**

Acquired resistance to sorafenib greatly limits its therapeutic efficiency in the treatment of hepatocellular carcinoma (HCC). Increasing evidence indicates that long noncoding RNAs (lncRNAs) play important roles in the resistance to anti-cancer drugs. The present study aims to explore the involvement of lncRNA SNHG1 (small nucleolar RNA host gene 1) in sorafenib resistance and how SNHG1 is associated with overexpressed microRNA-21 (miR-21) and the activated Akt pathway, which have been demonstrated to mediate this resistance in HCC cells.

**Methods:**

Sorafenib-resistant HCC (SR-HCC) cells were generated and their sorafenib-resistant properties were confirmed by cell viability and apoptosis assays. Potential lncRNAs were screened by using multiple bioinformatics analyses and databases. The expression of genes and proteins was detected by qRT-PCR, Western blot and in situ hybridization. Gene silencing was achieved by specific siRNA or lncRNA Smart Silencer. The effects of anti-SNHG1 were evaluated in vitro and in experimental animals by using quantitative measures of cell proliferation, apoptosis and autophagy. The binding sites of miR-21 and SNHG1 were predicted by using the RNAhybrid algorithm and their interaction was verified by luciferase assays.

**Results:**

The Akt pathway was highly activated by overexpressed miR-21 in SR-HCC cells compared with parental HCC cells. Among ten screened candidates, SNHG1 showed the largest folds of alteration between SR-HCC and parental cells and between vehicle- and sorafenib-treated cells. Overexpressed SNHG1 contributes to sorafenib resistance by activating the Akt pathway via regulating SLC3A2. Depletion of SNHG1 enhanced the efficacy of sorafenib to induce apoptosis and autophagy of SR-HCC cells by inhibiting the activation of Akt pathway. Sorafenib induced translocation of miR-21 to the nucleus, where it promoted the expression of SNHG1, resulting in upregulation of SLC3A2, leading to the activation of Akt pathway. In contrast, SNHG1 was shown to have little effect on the expression of miR-21, which downregulated the expression of PTEN, leading to the activation of the Akt pathway independently of SNHG1.

**Conclusions:**

The present study has demonstrated that lncRNA SNHG1 contributes to sorafenib resistance by activating the Akt pathway and its nuclear expression is promoted by miR-21, whose nuclear translocation is induced by sorafenib. These results indicate that SNHG1 may represent a potentially valuable target for overcoming sorafenib resistance for HCC.

**Electronic supplementary material:**

The online version of this article (10.1186/s13046-019-1177-0) contains supplementary material, which is available to authorized users.

## Background

Hepatocellular carcinoma (HCC) has the third highest rate of mortality worldwide [1]. It manifests frequently in patients with cirrhosis and liver dysfunction, limiting interventions with systemic cytotoxic drugs [2]. To date, four drugs targeting tyrosine kinases have been approved for the treatment of HCC. Lenvatinib has recently been used as a first-line drug [3], and regorafenib and cabozantinib have been approved as second-line drugs after the failure of sorafenib [4], but their use is not widely accepted. Sorafenib is the first approved systemic drug and continues to play the most important role in the management of advanced HCC [5]. However, the known resistance to sorafenib greatly minimizes its therapeutic benefits. Therefore, exploring the mechanisms for sorafenib resistance and seeking novel molecular targets are urgently required.

We have previously demonstrated that the Akt pathway is highly activated in sorafenib-resistant HCC (SR-HCC) cells and the inhibition of Akt could reverse this resistance by switching autophagy from a cytoprotective role to a death-promoting mechanism [6]. MicroRNAs (miRNAs) are a group of small noncoding RNAs, which regulate multiple cellular functions and have emerged as potential targets in the anti-cancer campaign [7]. In exploring the miRNA-related mechanisms that regulate the activation of Akt involved in sorafenib resistance, we found that miR-21 was highly expressed in SC-HCC cells and was able to activate the Akt pathway by dysregulating phosphatase and tensin homolog (PTEN) [8].

Long non-coding RNAs (lncRNAs) play crucial roles in controlling gene expression involving numerous biological processes in many diseases [9]. One of the mechanisms of their action involves the formation of regulatory networks with other RNA species, such as miRNAs and mRNAs [10, 11]. Several lncRNAs have been shown to participate in anti-cancer drug resistance [12]. For instance, lncARSR secreted by exosomes regulated sunitinib resistance by acting as competing endogenous RNAs for miR-34 and miR-449 in renal cancer [13], LncRNA MIR-100HG was involved in cetuximab resistance by regulating the wnt/β-catenin pathway via miR-100 and miR-125b in colorectal carcinoma [14], and LncRNA HULC reduced the sensitivity to cytotoxic drugs by stabilizing Sirt1-induced autophagy in HCC cells [15]. However, it is unknown whether lncRNAs also contribute to the mechanisms of sorafenib resistance in HCC cells.

LncRNA SNHG1, whose gene is located at chromosome 11q12.3, is expressed in the nucleus of most cells as well as in the cytoplasm in some cell-types [16]. SNHG1 is widely distributed in the body and participates in the regulation of proliferation, invasion and metastasis of many types of cancer cells including HCC [17–19]. Further, SNHG1 has been shown to be a poor survival indicator for HCC patients [19]. One of the reported mechanisms of SNHG1 for its biological action is the activation of the Akt pathway. It achieves this activation by promoting the transcription of the neighboring protein-coding gene, SLC3A2 (solute carrier family 3 member 2), *in cis* via binding the mediator complex to facilitate the establishment of enhancer-promoter interaction [20]. The Akt pathway is highly activated in SR-HCC cells [6, 21–23], thus it is speculated that SNHG1 may play a key mechanistic role in the resistance to sorafenib in HCC.

## Materials and methods

### Cells, antibodies, and reagents

Human HCC HepG2 and Huh7 cells, and SR-HCC cells (HepG2-SR and Huh7-SR cells established from parental HepG2 and Huh7 cells, respectively) have previously been described [6, 23, 24]. All cell lines were confirmed as negative for mycoplasma infection by using a PCR-based Universal Mycoplasma Detection kit (American Type Culture Collection, Manassas, VA, USA). Cells were routinely cultured in Dulbecco’s Modified Eagle Medium (DMEM) (Gibco BRL, Grand Island, NY, USA) supplemented with 10% fetal bovine serum in a humidified atmosphere of 5% CO_2_. The SR-HCC cells were kept by culturing them in the presence of sorafenib. Information for antibodies, reagents and kits is described in details under Additional file 1.

### Animal experiments

Male BALB/c-nu/nu mice (aging 6–8 weeks) obtained from SLAC laboratory Animal Co., Ltd. (Shanghai, China) were maintained at the Animal Research Center of the First Affiliated Hospital of Harbin Medical University. Animal experiments were performed as described previously [6, 23, 24], according to a permit (No. SYXK20020009, Harbin Medical University) in compliance with the Experimental Animal Regulations by the National Science and Technology Commission, China. Briefly, Huh7-SR cells (5 × 10^6^) were subcutaneously injected into mice receiving daily administration of sorafenib at a low dose of 10 mg/kg, which could help Huh7-SR cell maintain their sorafenib-resistant ability. Mice were monitored and the appearance of tumors recorded. 25 days later, mice bearing subcutaneous tumors (~ 100 mm^3^ in volume) were selected and randomly assigned to four treatment groups: control, sorafenib, anti-SNHG1 and sorafenib + anti-SNHG1. Sorafenib was suspended in an oral vehicle containing Cremophor (Sigma-Aldrich, Shanghai, China), 95% ethanol and water in a ratio of 1:1:6, and administered to mice in the sorafenib and sorafenib + anti-SNHG1 groups by gavage feeding at a dose of 30 mg/kg daily. Anti-SNHG1 was intratumorally delivered by means of lncRNA Smart Silencer mixed with Lipofectamine2000 (5 pmol/μl of oligonucleotides solution) once every 3 days for a total of five times in the anti-SNHG1 and sorafenib + anti-SNHG1 groups. Mice in the control group received oral vehicle and intratumoral injection of negative control (NC) oligonucleotides. 2 days following intratumoral injections, two mice from the control and anti-SNHG1 groups were sacrificed and tumors harvested for analysis. The remaining mice were further monitored for recording the size of tumors every 5 days and euthanized 21 days after treatments commenced.

#### In situ hybridization for detecting miR-21 and SNHG1

The in situ expression of miRNA and lncRNAs was detected by using previously described methods with appropriate modifications [25]. Double digoxigenin (DIG)-labelled locked nucleic acid probes for miR-21 (TCAACATCAGTCTGATAA GCTA, RNA-Tm 84 °C), SNHG1 (GTTCTCATTTTTCTACTGCTCGTG, RNA-Tm 85 °C), and a scrambled sequence (GTGTAACACGTCTATACGCCCA, RNA-Tm 87 °C) as a negative control (Exiqon, Vedbaek, Denmark) were used according to the manufacturer’s manual. Briefly, cells were fixed with 4% paraformaldehyde and incubated with Proteinase-K (15 μg/mL) for 10 min at 37 °C. After being washed twice in phosphate buffered saline (PBS), cells were dehydrated in ethanol, blocked in prehybridization buffer (3% bovine serum albumin [BSA]) for 30 min at 55 °C and incubated in hybridization buffer with probes (diluted at 1:2000) for 1 h at 55 °C. They were then washed with standard saline citrate buffer and blocked with 4% BSA for 1 h at room temperature. Positive signals were developed by an overnight incubation with an anti-DIG primary mouse monoclonal antibody at 4 °C, followed by incubations with fluorescein isothiocyanate (FITC)-conjugated or Cy3-conjugated secondary antibodies. DAPI (4′,6-diamidino-2-phenylindole) was used to stain cell nuclei. Stained cells were visualized by laser scanning confocal microscopy. For detecting SNHG1 expression in tumor sections, similar methods were applied except for the use of horseradish peroxidase (HRP)-conjugated secondary antibody, and immunoreaction with Sigma FAST DAB (3,3′-diaminobenzidine tetrahydrochloride) and CoCl_2_ enhancer tablets. All buffers in the above-described experiments were freshly prepared on the day of the experiment.

### Methodologies for assessing cell viability, apoptosis and autophagy, transfection of oligonucleotides and lncRNA smart silencer, cell fraction isolation, plasmid constructs, luciferase assays and gene expression detection in vitro and in vivo.

These were described in detail under Additional file 1 and have also been described previously [6, 23, 26]

### Statistical analysis

The data were expressed as mean values ± standard deviation (SD) from at least three independent experiments. Comparisons were made by using a one-way analysis of variance followed by a Dunnet’s t-test with the statistical software SPSS 18.0 for Windows (SPSS 224 Inc., IL, USA). *P* < 0.05 was considered significant.

## Results

### Sorafenib-resistant HCC cells overexpress MiR-21 and SNHG1

The sorafenib-resistant properties of SR-HCC cells were confirmed by comparing cell viability and apoptosis rates with parental HCC cells following incubation with various concentrations of sorafenib (Additional file 2: Figure S1).

We have previously reported that overexpressed miR-21 contributes to sorafenib resistance by activating the Akt pathway via downregulating PTEN, and anti-miR-21 could reverse this resistance in HCC cells [8]. In accord, the expression levels of miR-21 were significantly increased by sorafenib exposure in both parental and SR-HCC cells, and SR-HCC cells expressed higher levels of miR-21 than their parental sublines, in either the presence or absence of sorafenib (Additional file 2: Figure S2A). PTEN, a known miR-21 targeting gene in HCC cells [27], was downregulated in SR-HCC cells compared to parental sublines, and sorafenib incubation resulted in a significant reduction of PTEN expression in both parental and SR-HCC cells (Additional file 2: Figure S2B and C). In addition, transfection of miR-21 mimics downregulated PTEN expression in Huh7 cells that expressed lower levels of miR-21, while anti-miR-21 upregulated the expression of PTEN in Huh7-SR cells that expressed higher levels of miR-21 (Additional file 2: Figure S2 D and E).

Given that the interaction between lncRNAs and miRNAs plays important roles in gene regulation [28], we sought to identify potential lncRNAs that interact with miR-21 by studying multiple bioinformatics analyses and databases (http://starbase.sysu.edu.cn, www.lncrnadb.org, https://omictools.com, https://bioinfo.life.hust.edu.cn/lncRNASNP). Ten potential lncRNAs were screened based on the criteria of free energy <− 10 kcal/mol and score > 140 (Additional file 2: Table S1), and their expression levels were further detected by qRT-PCR with a panel of specific primers (Additional file 2: Table S2). Among the ten candidates, SNHG1 was shown to have the largest folds of alteration between SR-HCC and parental cells and between vehicle- and sorafenib-treated cells, and this change remained consistently elevated in both HepG2 and Huh7 cells (Additional file 2: Figure S3).

### Sorafenib induces nuclear retention of SNHG1 and miR-21 in HCC cells

**Fig. 1:**
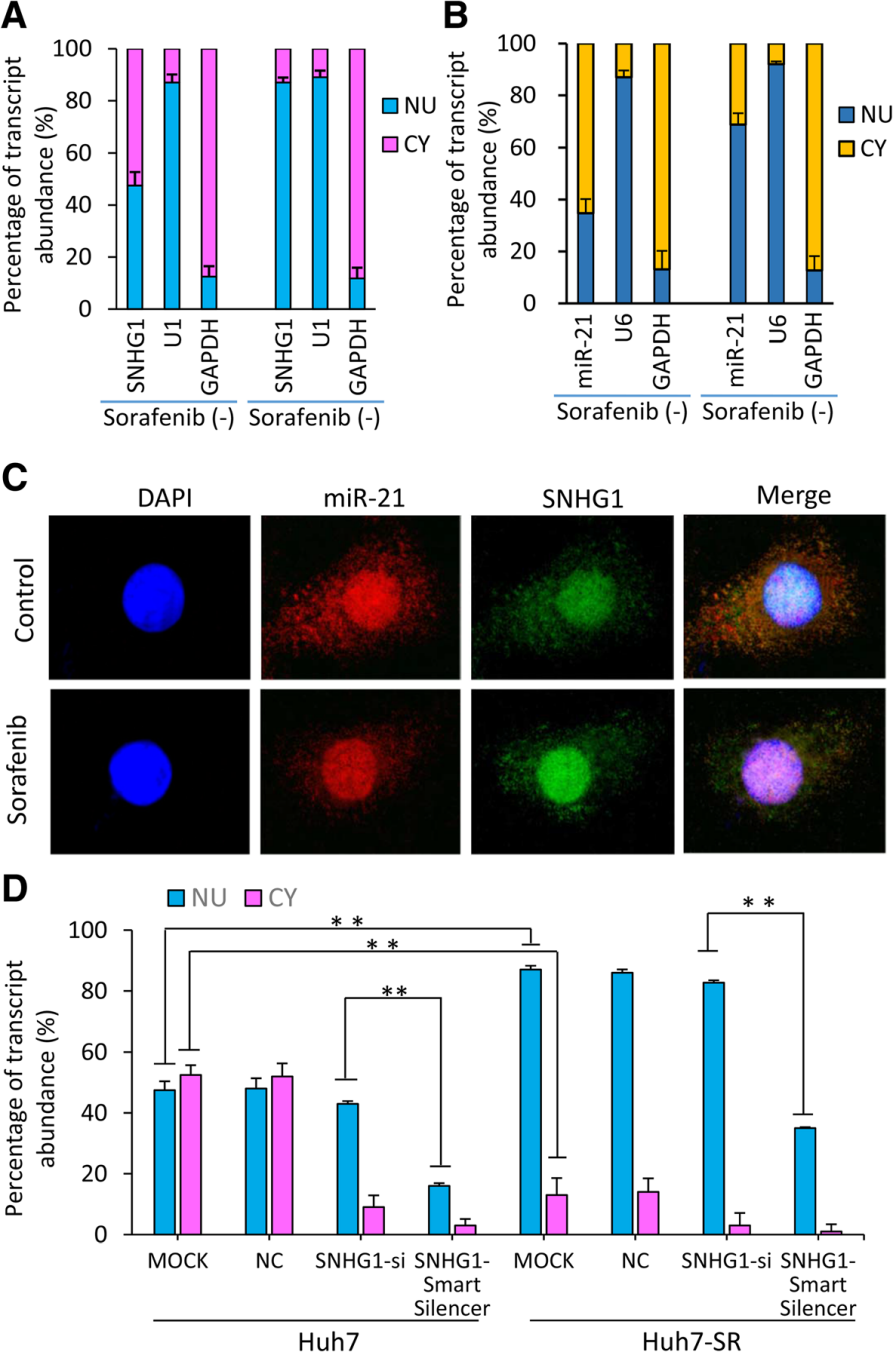
Sorafenib induces nuclear accumulation of miR-21 and SNHG1 that are overexpressed in sorafenib-resistant HCC cells. **a-c** Huh7 cells were incubated with sorafenib (2.5 μM) for 48 h. Total RNA was extracted from nuclear (NU) and cytoplasmic (CY) fractions, and the expression of SNHG1 (**a**) and miR-21 (**b**) was measured by qRT-PCR and normalized. U1 and U6 were used as internal nuclear controls for SNHG1 and miR-21, respectively. GAPDH was used as a cytoplasmic control. The results were expressed as means ± SD (*n* = 3). **c** The above cells were subjected to in situ hybridizations of miR-21 (5′-DIG tagged probe, identified with Cy3-conjugated Ab in red) and SNHG1 (5′-DIG tagged probe, identified with FITC-conjugated Ab in green), and stained with DAPI (blue). Three images from the same cell were merged. **d** Huh7 and Huh7-SR cells were untreated (mock) or transfected with negative control (NC), siRNA targeting SNHG1 (SNHG1-si) and SNHG1-smart Silencer for 24 h. The expression of SNHG1 was detected in nuclear and cytoplasmic fractions as above. “*” (*P* < 0.05) and “**” (*P* < 0.001) indicate a significant difference

We next examined intracellular location of SNHG1 and miR-21 by detecting their expression in nuclear and cytoplasmic fractions isolated from lysates of Huh7 cells that had been incubated in the absence or presence of sorafenib (2.5 μM) for 48 h. Nuclear and cytoplasmic fractions possessed 47.5 and 52.5% of total SNHG1, respectively, in untreated Huh7 cells, while sorafenib incubation led to a significant increase of SNHG1 in nuclear fraction (84.7%) (Fig. 1a), indicating that sorafenib may induce a nuclear accumulation of SNHG1 in HCC cells, in support of a previous study where doxorubicin induced SNHG1 nuclear retention [16]. Intriguingly, sorafenib also significantly increased the proportion of miR-21 in nuclear fractions to 68.6%, compared with 34.7% in untreated Huh7 cells (Fig. 1b). The change of nuclear retention of SNHG1 and miR-21 induced by sorafenib was visualized by in situ hybridization with specific probes in Huh7 cells (Fig. 1c). The nuclear proportion of SNHG1 was also significantly higher in Huh7-SR cells (79.6%) than parental sublines (Fig. 1d). Transfection of an siRNA targeting SNHG1 (SNHG-si) significantly reduced the expression levels of SNHG1 in cytoplasmic fractions, while transfection of SNHG1-Smart Silencer significantly reduced SNHG1 expression in both nuclear and cytoplasmic fractions, compared with mock-treated or NC-transfected cells (Fig. 1d). Therefore, SNHG1-Smart Silencer was used as an anti-SNHG1 tool in the following experiments.

### Overexpressed SNHG1 contributes to sorafenib resistance by activating the Akt pathway via regulating SLC3A2

**Fig. 2:**
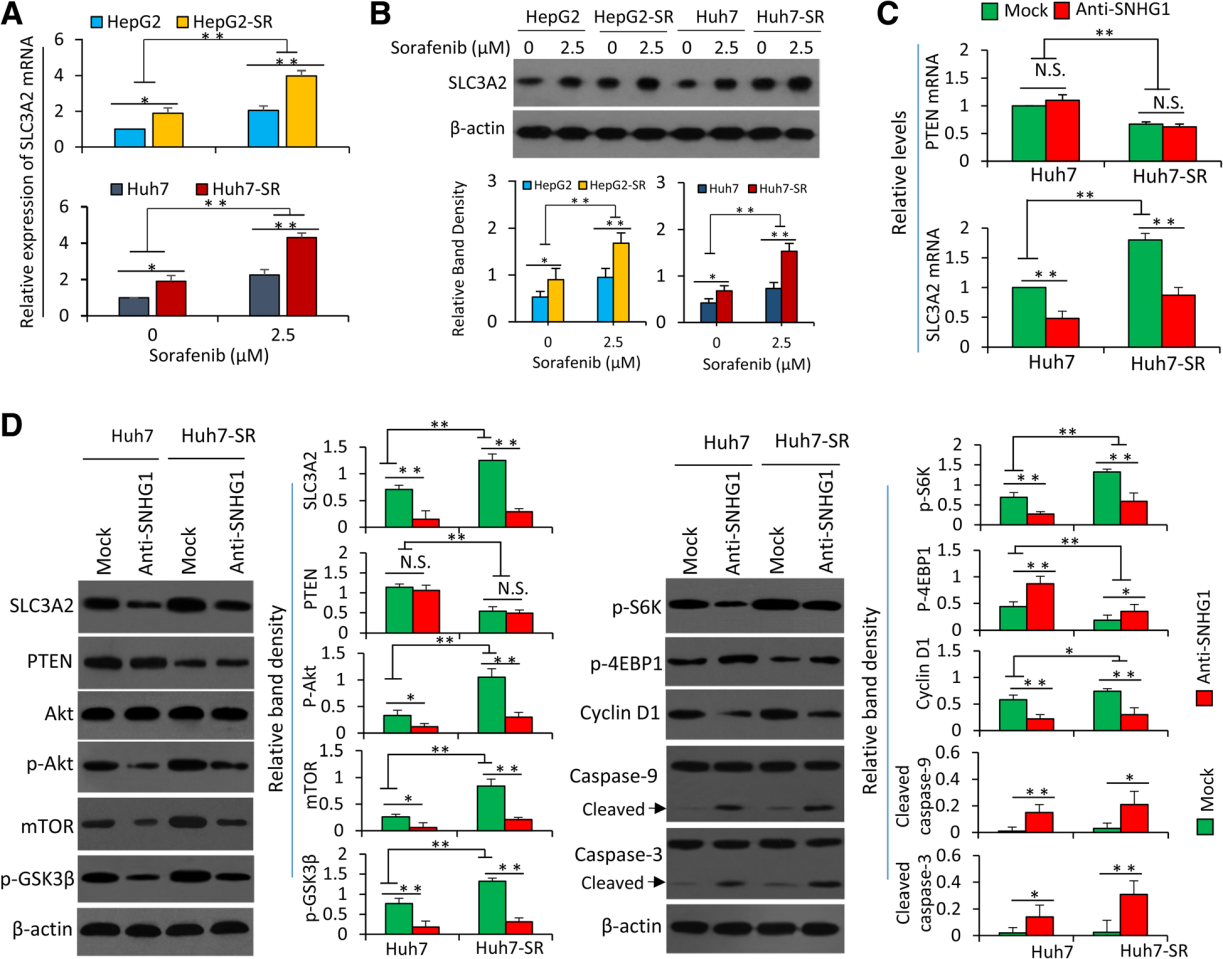
SNHG1 activates of the Akt pathway by regulating SLC3A2 in HCC cells. **a** and **b** HepG2, HepG2-SR, Huh7 and Huh7-SR cells were incubated with sorafenib (2.5 μM) for 48 h, and then subjected to qRT-PCR for detecting SLC3A2 mRNA (**a**) and Western blot analysis for detecting SLC3A2 protein (**b**). **c** and **d** Huh7 and Huh7-SR cells were untreated (mock) or transfected with SNHG1 Smart Silencer (anti-SNHG1) for 48 h. Cells were subjected to RT-PCT for detecting mRNA expressions (**c**) and Western blot analysis for detecting protein expressions (**d**). The mRNA expression level in untreated Huh7 cells was defined as 1. The density of Western blot band was normalized to β-actin. N.S., no significance. “*” (*P* < 0.05) and “**” (*P* < 0.001) indicate a significant difference

It has been reported that SNHG1 activates the Akt signaling pathway by promoting the transcription of SLC3A2 [20] and Akt is highly activated in SR-HCC cells [6]. We first examined the expression of SLC3A2 in HCC cells, which were incubated in the absence or presence of sorafenib (2.5 μM) for 48 h. SR-HCC cells expressed higher levels of SLC3A2 than their parental sublines, and sorafenib exposure upregulated its expression in both parental and SR-HCC cells (Fig. 2a and b). Depletion of SLC3A2 by an siRNA enhanced the activity of sorafenib in inhibiting the viability (Additional file 2: Figure S4A) and inducing apoptosis (Additional file 2: Figure S4B and C), and abolished the activation of Akt induced by sorafenib and enhanced the ability of sorafenib in inducing caspase-3 cleavage (Additional file 2: Figure S4D).

Anti-SNHG1 reduced the expression of SLC3A2 in both Huh7 and Huh7-SR cells (Fig. 2c and d). The Akt pathway was highly activated in Huh7-SR cells evidenced by the increased expression of p-Akt (ser473) and the sequential alterations of the downstream factors including mTOR (mTOR, mammalian target of rapamycin), p-GSK3β (phosphorylated glycogen synthase kinase 3β), p-S6K (phosphorylated ribosomal protein S6 kinase) and p-4EBP1 (phosphorylated eukaryotic translation initiation factor 4E-binding protein 1) (Fig. 2d), in consistent with our previous reports [6, 23]. Anti-SNHG1 treatment resulted in a significant downregulation of SLC3A2 and p-Akt, leading to the decreased expression of mTOR, p-GSK3β and p-S6K and increased expression of p-4EBP1 in both Huh7 and Huh7-SR cells (Fig. 2d). Inhibition of the Akt pathway by anti-SNHG1 also downregulated cyclin D1 and promoted the cleavage of caspase-9 and sequential caspase-3 (Fig. 2d). PTEN, a well-known upstream regulator that directly inactivates the Akt pathway [29], was downregulated in SR-HCC cells (Fig. 2a and b), in consistent with our previous study [8]. However, Anti-SNHG1 had little effects on the expression of PTEN mRNA or protein (Fig. 2c and d), indicating that SNHG1 may regulate the activation of Akt pathway independent of PTEN.

### Depletion of SNHG1 enhances the activity of sorafenib to induce the apoptosis and autophagy of sorafenib-resistant HCC cells

**Fig. 3:**
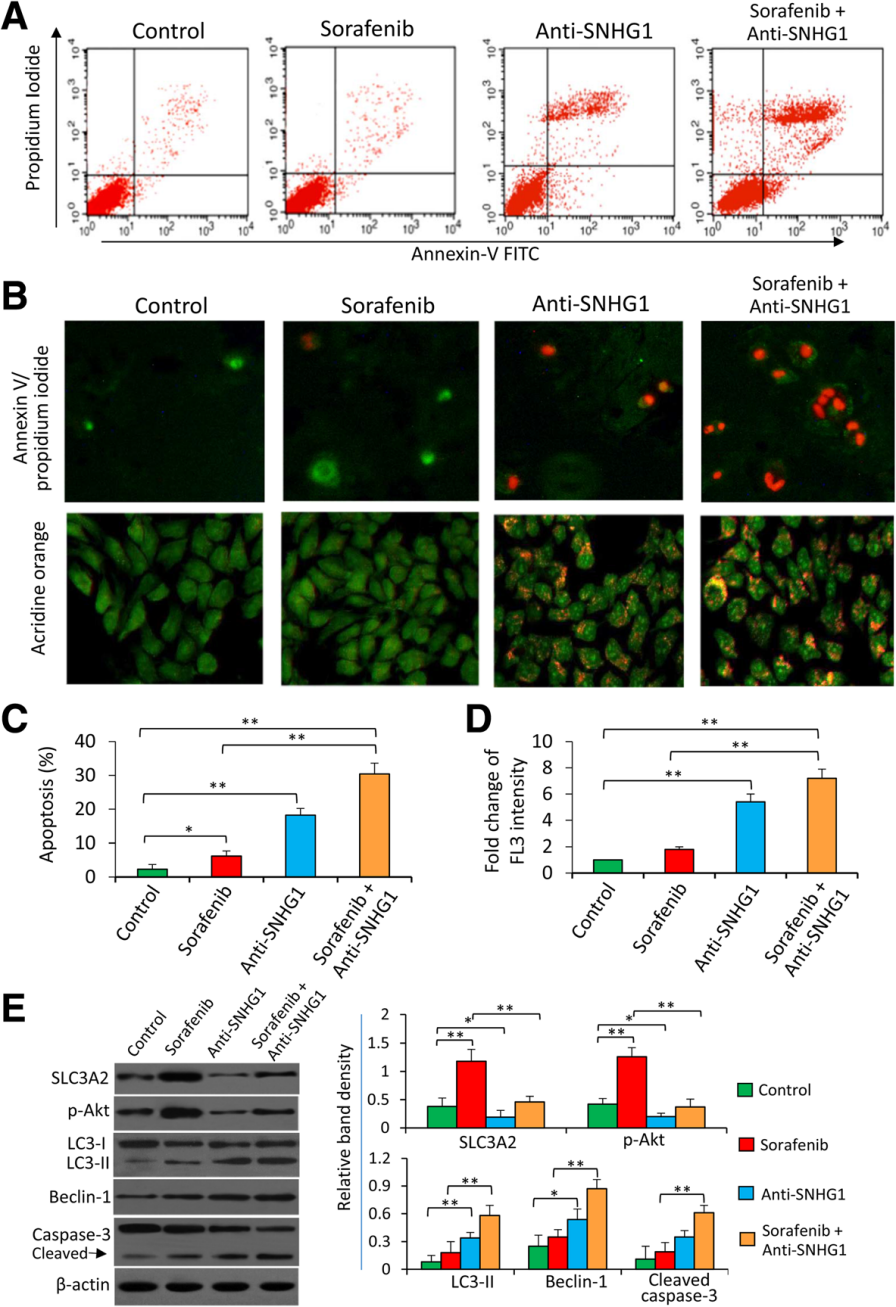
Depletion of SNHG1 enhances the effects of sorafenib in promoting apoptosis and autophagy of sorafenib-resistant cells. Huh7-SR cells either untreated (control) or transfected with SNHG1 Smart Silencer (anti-SNHG1) were incubated for 48 h in the absence or presence of sorafenib (5 μM). **a** Representative dot plots were from the above cytometrically analyzed cells. **b** Representative images were taken from the above cells stained by Annexin V/propidium iodide or acridine orange (magnification × 200). **c** Cell apoptosis (%) was measured by cytometry. **d** The fold change of acridine orange fluorescence intensity (FL3) versus the untreated controls, which was defined as 1, was calculated. **e** Cells were subjected to Western blot analysis. The density of each band was normalized to β-actin. “*” (*P* < 0.05) and “**” (*P* < 0.001) indicate a significant difference

Mock- and anti-SNHG1-treated SR-HCC cells were incubated with various concentrations of sorafenib for 48 h. Anti-SNHG1 inhibited the viability and promoted the apoptosis of SR-HCC cells in either the presence or absence of sorafenib (Additional file 2: Figure S5). Specifically, both sorafenib and anti-SNHG1 induced cell apoptosis, but anti-SNHG1 exhibited a stronger pro-apoptotic activity than sorafenib, and anti-SNHG1 enhanced the pro-apoptotic ability of sorafenib (Fig. 3a-c). We have previously demonstrated that autophagy plays a death-promoting role in SR-HCC cells [6, 8]. Here we showed that anti-SNHG1 treatment resulted in significantly more acridine orange-stained acidic vesicular organelles (AVOs), while sorafenib only induced a slight increase of AVOs (Fig. 3b). Quantitative analysis confirmed that anti-SNHG1-transfected cells had significantly higher fluorescence intensity (FL3) and the combination of sorafenib and anti-SNHG1 led to even higher FL3 (Fig. 3d). In terms of mechanisms, anti-SNHG1 corrected the enhanced activation of Akt by sorafenib, and led to the increased conversion of LC3 (microtubule-associated protein 1 light chain 3)-I to LC3-II, upregulation of Beclin-1, and an increased cleavage of caspase-3 (Fig. 3e).

### Depletion of SNHG1 suppresses sorafenib-resistant tumors in vivo

**Fig. 4:**
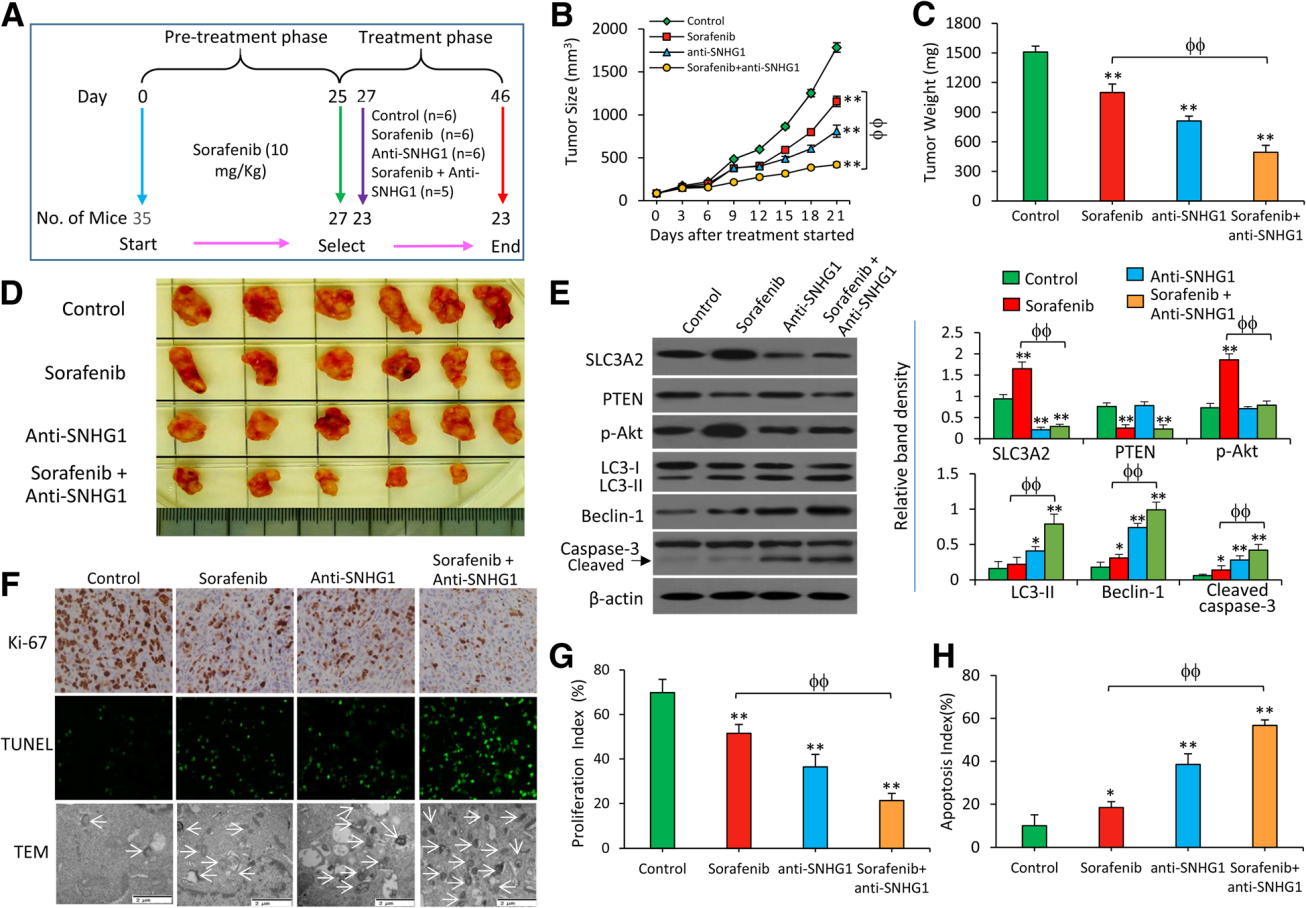
Depletion of SNHG1 enhances the efficacy of sorafenib to suppress sorafenib-resistant HCC tumors in vivo. **a** Animal experimental schedule was described as in Materials and Methods. **b** The size (mm^3^) of tumors was recorded. **c**and **d**) Tumors harvested at the end of experiments were weighed (**c**) and photographed (**d**). **e** Tissue homogenates were Western blotted. The density of each band was normalized to β-actin. **f** Representative images of tumor sections were immunostained with an anti-Ki67 Ab and TUNEL. **g** Proliferation index (%) and **h** apoptosis index (%) were quantified. “*” (*P* < 0.05) and “**” (*P* < 0.001) indicate a significant difference from controls. “ϕ” (*P* < 0.05) and “ϕϕ” (*P* < 0.001) indicate a significant difference

Sorafenib-resistant tumors were established as shown in Materials and Methods and also as described previously [6, 23, 24]. 25 days after cell inoculation, 27 mice (27/35) bearing palpable tumors of ~ 100 mm^3^ in volume were selected and randomly assigned to four groups, which received respective treatments (Fig. 4a). Four mice from the control (*n* = 2) and anti-SNHG1 (*n* = 2) groups were sacrificed 2 days after the commencement of treatments, and depletion of SNHG1 by anti-SNHG1 was confirmed by in situ hybridization (Additional file 2: Figure S6A). SR-HCC tumors were shown to be refractory to sorafenib since sorafenib-treated tumors (1157.4 ± 60.6 mm^3^ in volume, 1097.3 ± 86.8 mg in weight) were only slightly smaller than control tumors (1783.8 ± 55.6 mm^3^ in volume, 1509.1 ± 60.2 mg in weight) 21 days after the commencement of treatments (Fig. 4b-d). However, anti-SNHG1 treatment significantly reduced the size of tumors (811.3 ± 70.5 mm^3^ in volume, 812.3 ± 48.7 mg in weight) by 54.5%, and the combinational therapy resulted in a further reduction of tumor size (420.0 ± 32.2 mm^3^ in volume, 494.3 ± 70.6 mg in weight) by 76.4%, compared with control tumors at the end of experiments (Fig. 4b-d).

The expression of the key proteins in the Akt pathway was analyzed by Western blot analysis (Fig. 4e) and immunohistochemistry (Additional file 2: Figure S6B). In consistent with the in vitro results (Fig. 3e), anti-SNHG1 was shown to correct the upregulation of SLC3A2 and the activation of Akt pathway induced by sorafenib, and combined with sorafenib to upregulate the expression of LC3-II, Belcin-1 and cleaved caspase-9/- 3 (Fig. 4e). Tumors were examined for in situ cell proliferation by immunohistochemistry with an anti-Ki67 Ab, apoptosis by TUNEL staining, and autophagy by electronic microscopy (Fig. 4f). Again, sorafenib had a weak inhibitory effect against cell proliferation and weak activities to induce apoptosis and autophagy in SR-HCC tumors. Anti-SNHG1 significantly reduced cell proliferation, and induced apoptosis and autophagy in situ. Quantitatively, anti-SNHG1 enhanced the effects of sorafenib in inhibiting cell proliferation and promoting apoptosis, since tumors treated with the combinational therapy had a further reduction in proliferation index and a further increase in apoptosis rate, compared with either sorafenib or anti-SNHG1 monotherapy (Fig. 4g and h).

### The regulatory effect between SNHG1 and miR-21 is in a one-way manner

**Fig. 5:**
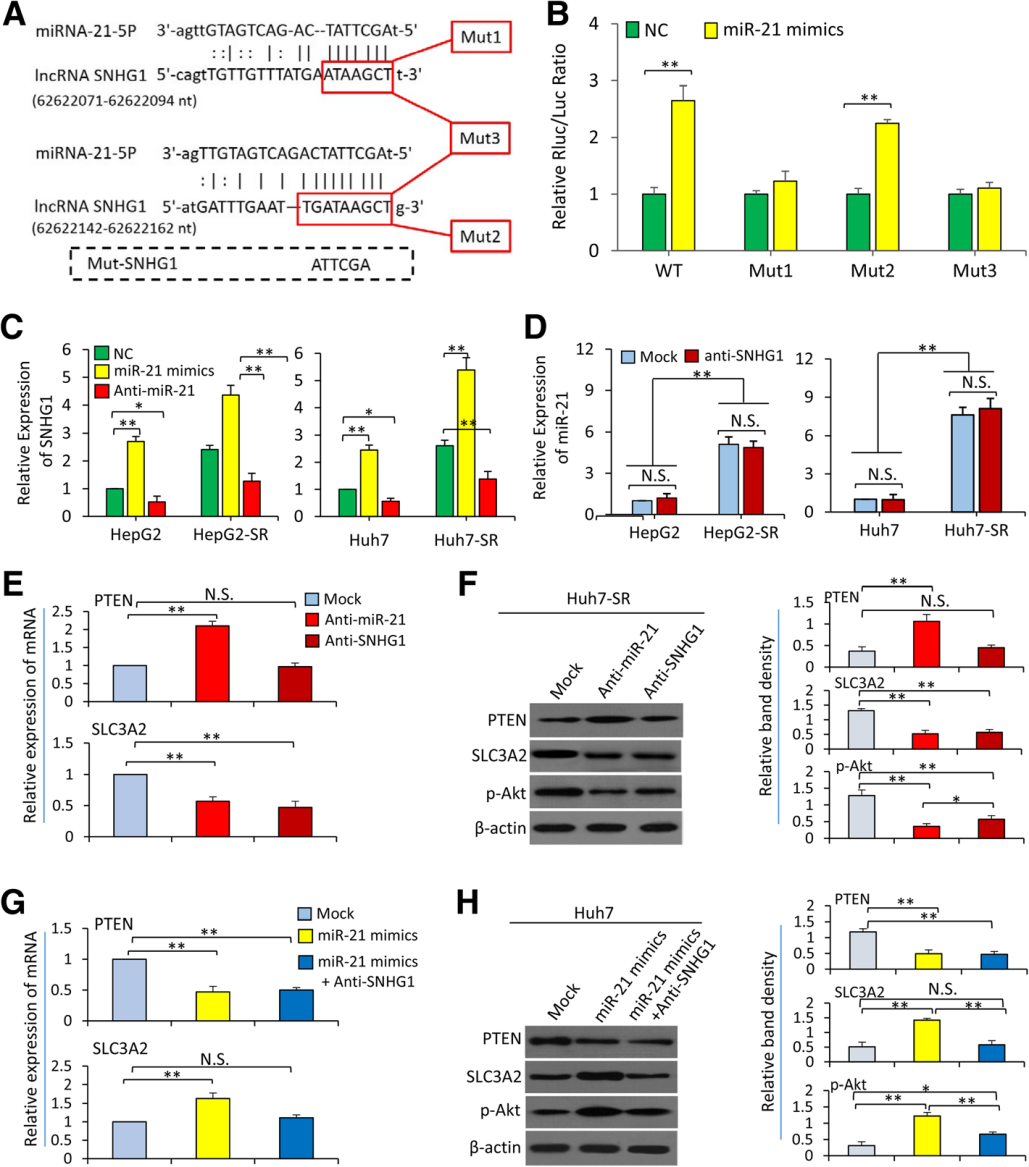
MiR-21 activates the Akt pathway partially by upregulating SNHG1 in HCC cells. **a** The binding sites between SNHG1 and miR-21 were predicted and 3 forms of mutated sequences (Mut1, Mut2 and Mut3) designed. **b** MiR-21 mimics or negative control (NC) oligonucleotides were co-transfected with a wild-type or mutated forms of SNHG1 into Huh7 cells. Cellular relative luciferase activities were measured. The luciferase activity in NC-transfected cells was defined as 1. **c** HepG2, HepG2-SR, Huh7 and Huh7-SR cells were transfected with NC, miR-21 mimics or anti-miR-21 for 48 h, and the expression levels of SNHG1 were measured by qRT-PCR, and the expression level of SNHG1 in NC-transfected respective parental cells was defined as 1. **d** The above cells were either untreated (mock) or transfected with SNHG1 Smart Silencer (anti-SNHG1) for 48 h, and the expression levels of miR-21 were measured by qRT-PCR, and the expression level of miR-21 in mock-treated respective parental cells was defined as 1. (**e** and **f**) Huh7-SR cells were either untreated (mock) or transfected with anti-miR-21 or anti-SNHG1 for 24 h, and subjected to qRT-PCR (**e**) and Western blot (**f**) analyses. (**g** and **h**) Huh7 cells were either untreated (mock) or transfected with miR-21 mimics or miR-21 mimics + anti-SNHG1 for 48 h, and subjected to qRT-PCR (**g**) and Western blot (**h**) analyses. The expression level of mRNA in mock-treated cells was defined as 1 in qRT-PCR analyses. N.S., no significance. “*” (*P* < 0.05) and “**” (*P* < 0.001) indicate a significant difference

The above results showed that both SNHG1 and miR-21 could activate the Akt pathway in HCC cells, we thus investigated their interactive effects. Two putative miR-21 binding sites were identified in the enhancer/promoter regions of SNHG1 gene at 62622071-62622094 nt and 62,622,142-62,622,162 nt by using the RNAhybrid algorithm (Fig. 5a). A pmiR-RB-REPORT™ dual luciferase reporter system was employed to determine whether the predicted binding sites were involved in SNHG1 regulation. An expression vector containing the enhancer region of wild-type SNHG1 (WT) and three vectors containing mutants at 3′-UTR (Mut1, “TAAGCT” [62622089–62,622,094] mutated to “ATTCGA”; Mut2, “TAAGCT” [62622157–62,622,162] mutated to “ATTCGA”; Mut3, the combination of Mut1 and Mut 2) were constructed (Fig. 5a). MiR-21 mimics and the above vectors were co-transfected into Huh7 cells, and the relative luciferase activities were measured. Transfection of miR-21 mimics resulted in significantly higher luciferase activities than NC oligonucleotides in Huh7 cells, which were co-transfected with WT vector (Fig. 5b). However, this difference was abolished by co-transfection with Mut1 or Mut3 vector, but not by co-transfection with Mut2 vector (Fig. 5b), indicating that miR-21 may promote the expression of SNHG1 through the binding of the sequence “ATTCGA” in miR-21 to the corresponding “TAAGCT” at 3′-UTR of SNHG1 (Fig. 5a). In support, miR-21 mimics significantly elevated, while anti-miR-21 significantly reduced, the expression level of SNHG1 in both parental and SR-HCC cells (Fig. 5c). However, downregulation of SNHG1 by anti-SNHG1 had little effects on the expression levels of miR-21 in either parental or SR-HCC cells (Fig. 5d). The above results indicate that the regulatory effects between miR-21 and SNHG1 may be in a one-way manner. In order to further validate this point, we transfected Huh7-SR cells, which expressed higher levels of miR-21 and SNHG1 (Additional file 1: Figures S2 and S3), with miR-21 and anti-SNHG1. As shown in Fig. 5e and f, anti-miR-21 significantly upregulated the expression of PTEN, leading to the downregulation of p-Akt, while anti-SNHG1 had little effect on PTEN expression, but could downregulate the expression of SLC3A2, resulting in the downregulation of p-Akt. In addition, Huh7 cells, which expressed lower levels of miR-21 and SNHG1 (Additional file 2: Figures S2 and S3), were transfected with miR-21 mimics and anti-SNHG1. MiR-21 mimics downregulated the expression of PTEN, leading to the upregulation of p-Akt, and co-transfection of anti-SNHG1 could not abolish this effect of miR-21 mimics (Fig. 5g and h), indicating that miR-21 displays its regulatory effects on PTEN expression and Akt activation independent of SNHG1. However, miR-21 mimics upregulated the expression of SLC3A2, leading to the upregulation of p-Akt, and anti-SNHG1 could abolish this effect of miR-21 on SLC3A2 and p-Akt (Fig. 5g and h), indicating that miR-21 exhibits its effects on the SLC3A2/Akt pathway through regulating SNHG1.

## Discussion

Sorafenib occupies a unique position in the campaign against HCC because it is the most widely used systemic drug approved by many international and national authorities. However, sorafenib has demonstrated limited survival benefits, and some HCC patients initially respond to sorafenib but eventually surrender to disease progression [3]. Therefore, it is essential to investigate underlying mechanisms for the acquired insensitivity to sorafenib. In the present study, we show that sorafenib-induced upregulation of lncRNA SNHG1 contributes to this resistance by promoting the activation of the Akt pathway via regulating SL3A2. Depletion of SNHG1 enhanced the anti-cancer activities of sorafenib against SR-HCC cells that were refractory to sorafenib-induced proliferation inhibition and apoptosis in vitro and in animal experiments. Many studies have revealed that the Akt pathway is highly activated in SR-HCC cells [6, 8, 21, 22, 27, 30] and we have previously reported that overexpressed miR-21 activates the Akt pathway in SR-HCC cells [8]. It is well-known that the cross-talk between lncRNAs and miRNAs participates in many cellular biological processes [28]. Therefore, in the present study, we further explored the interaction between SNHG1 and miR-21. Intriguingly our study has revealed a novel regulatory pathway, in which sorafenib induces the translocation of miR-21 into the nucleus, where miR-21 binds to the enhancer region of SNHG1 and positively regulates its expression. Our results indicate that SNHG1 may be a novel downstream target of miR-21, and an important contributor of sorafenib resistance in HCC cells.

**Fig. 6:**
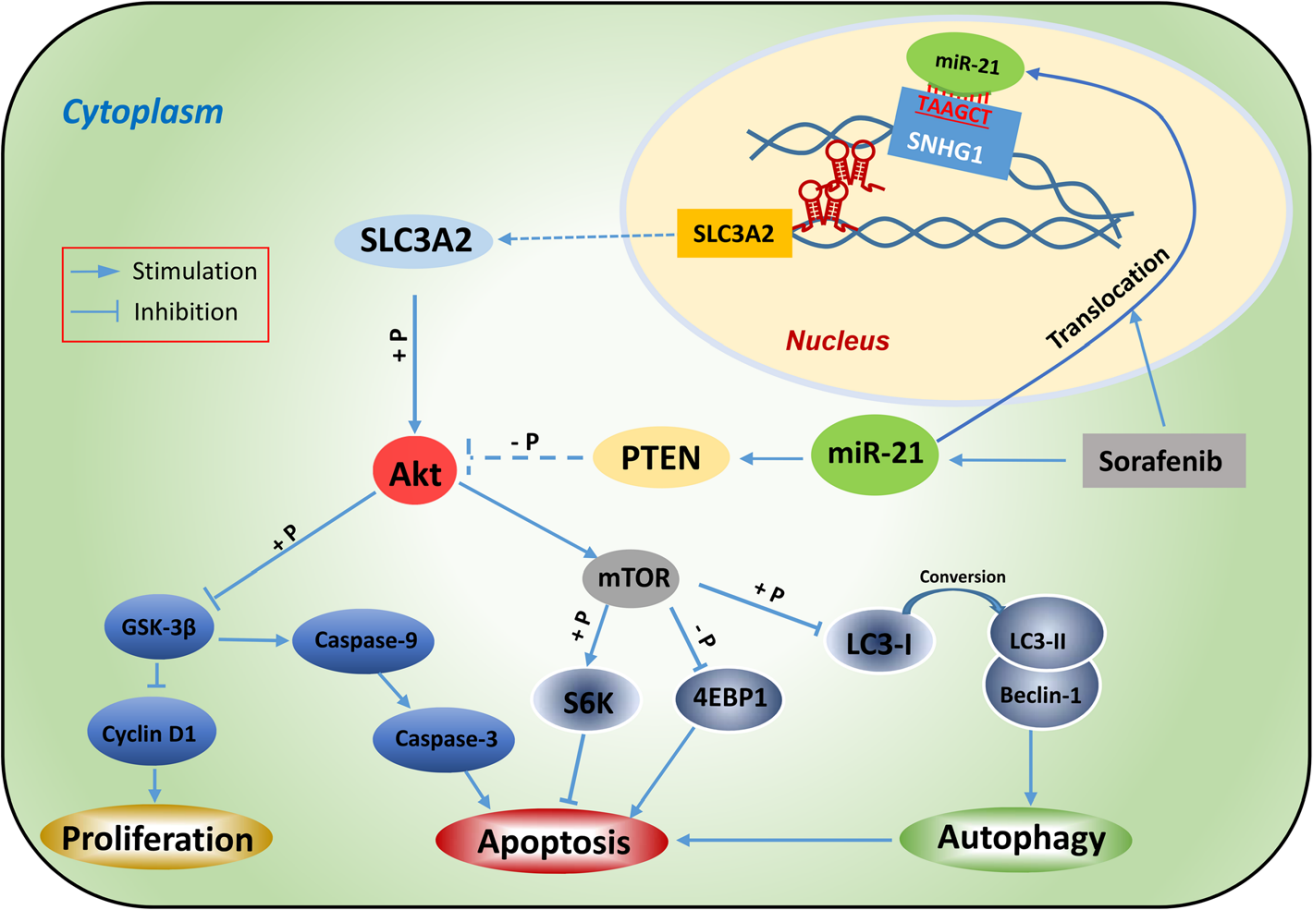
A proposed model of participation of SNHG1 in the regulation of signaling pathways that contribute sorafenib resistance in HCC cells. “→” indicates positive regulation or activation; “⊥”, negative regulation or blockade. “+P” indicates the enhancement of protein phosphorylation, while “-P”, the reduction of protein phosphorylation. A dotted line indicates the mechanisms investigated in previous studies, while a solid line, the mechanisms investigated in the present study. Abbreviations: 4EBP1, eukaryotic translation initiation factor 4E-binding protein 1; GSK-3β, glycogen synthase kinase 3β; LC3, microtubule-associated protein 1 light chain 3; mTOR, mammalian target of rapamycin; PTEN, phosphatase and tensin homolog; S6K, ribosomal protein S6 kinase; miR-21, microRNA-21; SLC3A2, solute carrier family 3 member 2; SNHG1, small nucleolar RNA host gene 1

The proposed mechanisms depicting the manner by which SNHG1 activates the Akt pathway and its regulation by miR-21 in HCC cells are shown schematically in Fig. 6. Sorafenib induces the upregulation of miR-21 and its translocation into the nucleus, where miR-21 positively regulates the expression of SNHG1 through binding to the sequence “ATTCGA” in the promoter region of SNHG1. SNHG1 promotes the neighboring transcription of the protein-coding gene SLC3A2 *in cis* by binding to the mediator complex [20], and the resulting overexpression of SLC3A2 induces the activation of the downstream Akt signaling pathway [31]. On the other hand, sorafenib induces overexpression of miR-21 [8], which in turn activates the Akt pathway by targeting PTEN, a well characterized tumor-suppressing phosphatase that inhibits Akt activation [29, 32]. Akt activation increases the phosphorylation of GSK3β, which downregulates the expression of cell proliferation protein cyclin D1 and induces the caspase cascade of apoptosis [29]. The activation of Akt also leads to upregulation and activation of mTOR [6], which controls autophagy by inhibiting the conversion of LC3-I to LC3-II, which is critical for cell autophagy [33, 34], and also regulates apoptotic proteins S6K and 4EBP1 [29]. In SR-HCC cells, autophagy switches from a cytoprotective factor to a death-promoting one, thus enhanced autophagy displays a pro-apoptosis function [6]. In support, the role of autophagy has been regarded as a double-edged sword in the anti-cancer activities of sorafenib against HCC [35, 36].

The interaction between noncoding RNAs is a novel and crucial regulatory mechanism helping them to achieve their diverse complex functions [37]. The best characterized mechanism for the interaction of lncRNAs and miRNAs pertains to the role of competitive endogenous lncRNAs, which serve as “sponges” for miRNAs [38]. On the other hand, miRNAs can regulate lncRNAs via a RNA-induced silencing complex, which often takes places in the cytoplasm [39]. In the canonical way, miRNAs are egressed from the nucleus and eventually mature in the cytoplasm, where they exert the post-transcriptional gene regulatory roles [40]. However, recent evidence suggests that certain mature cytoplasmic miRNAs can shuttle back to the nucleus, where they regulate gene expression [41–43]. The presence of mature miRNAs in the nucleus has recently been confirmed as a general phenomenon in mammalian cells [39, 41]. MiR-21 is the first miRNA with both a cytoplasmic and nuclear location in HeLa cells, with approximately 20% of mature miR-21 in the nucleus [44]. In agreement, the present study shows that 34.7% of miR-21 resides in nuclei of untreated Huh7 cells, which is supported by our microscopic evidence from in situ hybridization analysis. More interestingly, sorafenib induced the nuclear translocation of miR-21, which contributes to sorafenib resistance by upregulating SNHG1 in HCC cells.

MiR-21 is one of the few widely studied miRNAs expressed in many types of malignances including HCC, and plays a central role in cancer progression [27, 45]. The proposed “onco-miR addition” concept has further strengthened its role in carcinogenesis [45]. MiR-21 has also been identified as a key miRNA associated with drug insensitivity including the resistance to sorafenib [46]. In the conventional way, miR-21 exhibits its function by regulating downstream mRNAs [47]. The present study provides strong evidence that miR-21 could re-enter the nucleus, where it binds to the enhancer/promoter region of lncRNA SNHG1, leading to enhanced expression of SNHG1. This novel regulatory function of miRNAs on lncRNAs has been demonstrated in a previously reported study, where mature miR-140 trafficking in the nucleus can interact with lncRNA NEAT1, resulting in an increase in NEAT1 expression [48].

One weak point of the present study is that the results obtained in cell and animal experiments have not been validated in tumor tissues collected from HCC patients who have developed sorafenib resistance. The guideline of American Association for the Study of Liver Diseases (AASLD) indicates that sorafenib therapy is only approved for treating advanced HCC patients, judged clinically unsuitable for curative therapies including surgery and liver transplantation [49]. Due to the anatomic feature, clinical HCC tissues can be obtained by laparotomy, laparoscopy or fine-needle biopsy. However, the chance for harvesting SR-HCC tissues from late-staged patients is rare because such patients are unable to benefit from these invasive procedures in the view of stringent human ethic requirements, the informed consent and potentially risky complications. Postmortem may be another possible procedure for collecting SR-HCC tissues, but the quality of such tissues is hard to control. However, further validation in clinical SR-HCC tissues should be investigated in the future when such tissues are available since it will increase the clinical relevance and translation of the present results.

## Conclusions

The present study has demonstrated that lncRNA SNHG1 contributes to sorafenib resistance by activating the Akt pathway through upregulating SLC3A2 and its nuclear overexpression is promoted by miR-21 in HCC cells. Depletion of SNHG1 inhibited the activation of the Akt pathway, enhancing the efficacy of sorafenib in suppressing SR-HCC cells by promoting apoptosis and autophagy in cultured cells and in animal experiments. Despite the fact that two putative miR-21 and SNHG1 binding sites exist, SNHG1 was shown to be incapable of regulating miRNA expression, which is different from the well characterized role of lncRNAs as sponges for miRNAs. On the other hand, miR-21 did not exhibit a negative regulatory effect on SNHG1, which usually takes place in the cytoplasm via a miRNA-induced silencing complex. However, miR-21 was able to promote the expression of SNHG1 in the nucleus, to which the shuttling of miR-21 was induced by sorafenib in HCC cells. The present study has uncovered a novel regulatory pathway by which miR-21 promotes the expression of SNHG1, leading to the activation of Akt pathway. Our results also suggest that SNHG1 may represent a potentially valuable target for overcoming sorafenib resistance in HCC.

## Additional files

Additional file 1: Supplementary Materials and Methods. (PDF 149 kb)

Additional file 2: Supplementary tables and figures. (PDF 149 kb)

## Abbreviations

4EBP1: Eukaryotic translation initiation factor 4E-binding protein 1
ANOVA: Analysis of variance
GSK-3β: glycogen synthase kinase 3β
HCC: Hepatocellular carcinoma
LC3: Microtubule-associated protein 1 light chain 3
lncRNA: Long non-coding RNA
miR-21: microRNA-21
mTOR: mammalian target of rapamycin
NC: Negative control
PTEN: Phosphatase and tensin homolog
qRT-PCR: quantitative reverse-transcription polymerase chain reaction
S6K: ribosomal protein S6 kinase
SLC3A2: solute carrier family 3 member 2
SNHG1: small nucleolar RNA host gene 1
SR-HCC: sorafenib-resistant HCC
