# Correction to: LncRNA SNHG1 contributes to sorafenib resistance by activating the Akt pathway and is positively regulated by miR-21 in hepatocellular carcinoma cells

**DOI:** 10.1186/s13046-021-02183-3

**Published:** 2021-12-01

**Authors:** Weidong Li, Xuesong Dong, Changjun He, Gang Tan, Ziyi Li, Bo Zhai, Jing Feng, Xian Jiang, Chang Liu, Hongchi Jiang, Xueying Sun

**Affiliations:** 1 The Hepatosplenic Surgery Center, The First Affiliated Hospital of Harbin Medical University, Harbin, 150001 China; 2 Department of General Surgery, The Fourth Affiliated Hospital of Harbin Medical University, Harbin, 150001 China; 3 Department of Surgery, The Third Affiliated Hospital of Harbin Medical University, Harbin, China

**Correction to: J Exp Clin Cancer Res 38, 183 (2019)**

**https://doi.org/10.1186/s13046-019-1177-0**

Following publication of the original article [1], the authors identified some minor errors in Figs. 3 and 4, specifically:

- In Fig. 3a, the cytometrical dot plots originally contained errors
- In Fig. 4f, an incorrect image was originally used for the anti-SNHG1-treated tumor sections (1^st^ row, 3^rd^ column)

The authors provided the Journal with the original data. The corrected figures are given here. The corrections do not have any effect on the final conclusions of the paper. The original article has been corrected.

**Fig. 3:**
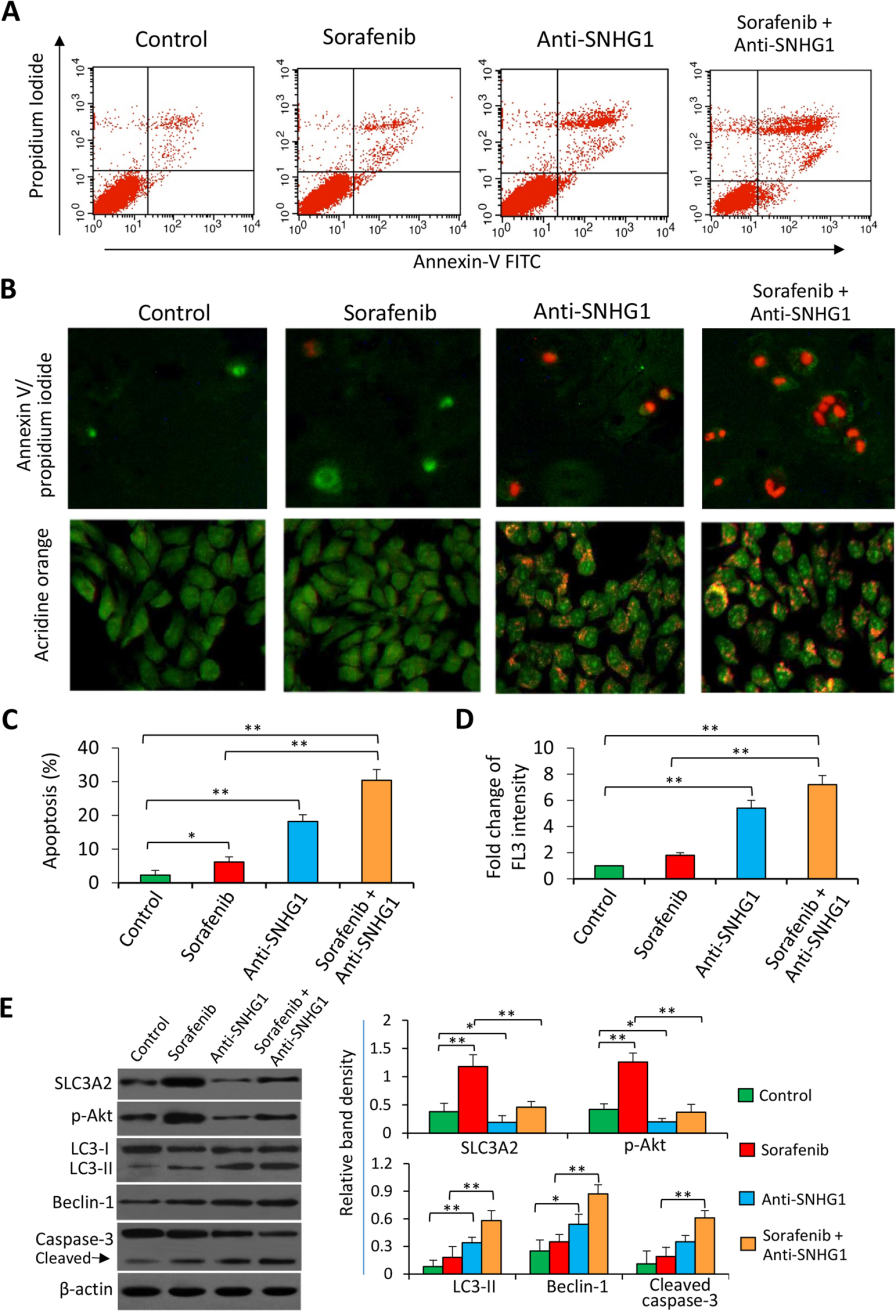
Depletion of SNHG1 enhances the effects of sorafenib in promoting apoptosis and autophagy of sorafenib-resistant cells. Huh7-SR cells either untreated (control) or transfected with SNHG1 Smart Silencer (anti-SNHG1) were incubated for 48 h in the absence or presence of sorafenib (5 μM). **a** Representative dot plots were from the above cytometrically analyzed cells. **b** Representative images were taken from the above cells stained by Annexin V/propidium iodide or acridine orange (magnification × 200). **c** Cell apoptosis (%) was measured by cytometry. **d** The fold change of acridine orange fluorescence intensity (FL3) versus the untreated controls, which was defined as 1, was calculated. **e** Cells were subjected to Western blot analysis. The density of each band was normalized to β-actin. “*” (*P* < 0.05) and “**” (*P* < 0.001) indicate a significant difference

**Fig. 4:**
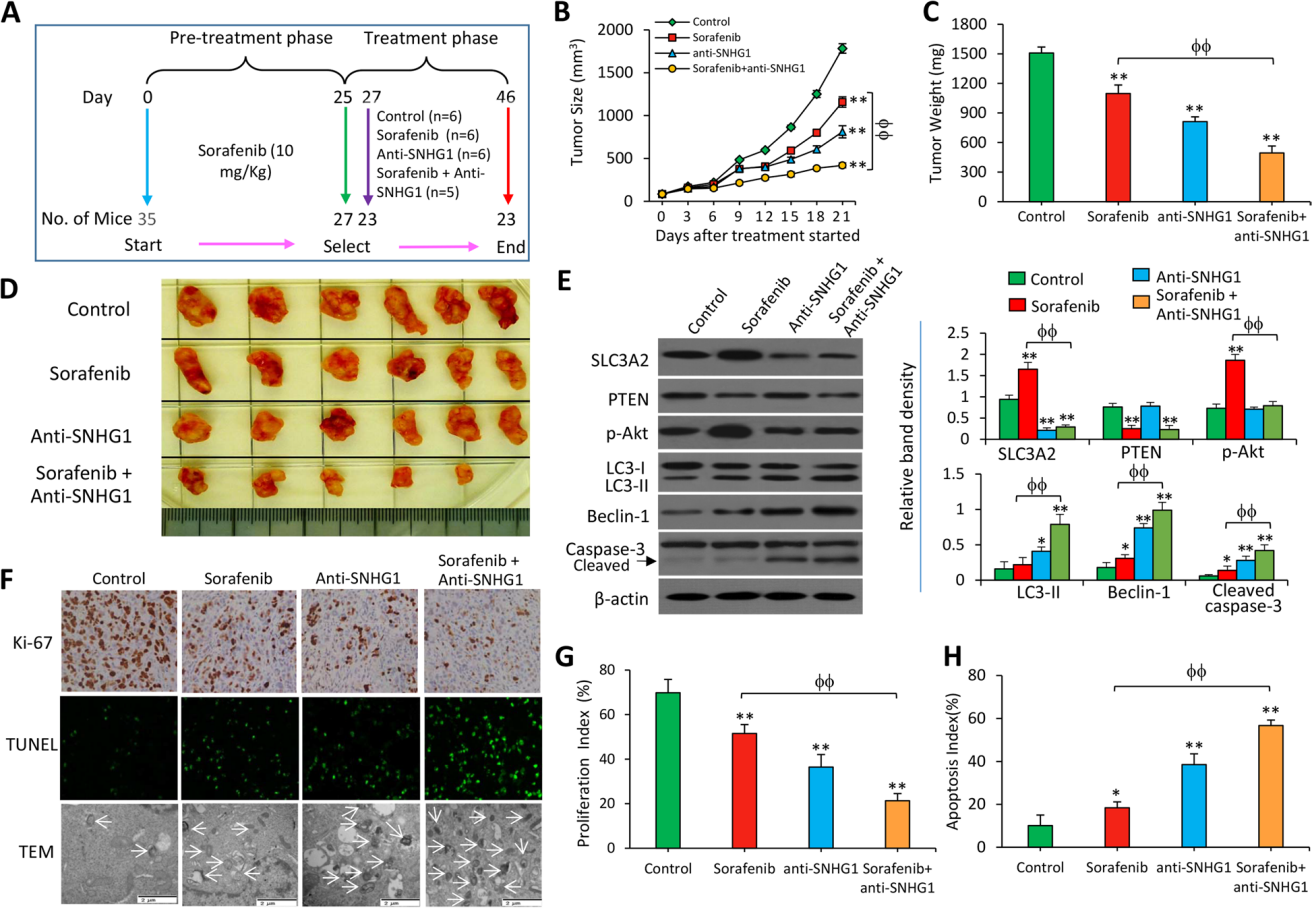
Depletion of SNHG1 enhances the efficacy of sorafenib to suppress sorafenib-resistant HCC tumors in vivo. **a** Animal experimental schedule was described as in Materials and Methods. **b** The size (mm3) of tumors was recorded. **c** and **d** Tumors harvested at the end of experiments were weighed (**c**) and photographed (**d**). **e** Tissue homogenates were Western blotted. The density of each band was normalized to β-actin. **f** Representative images of tumor sections were immunostained with an anti-Ki67 Ab and TUNEL. **g** Proliferation index (%) and h apoptosis index (%) were quantified. “*” (*P* < 0.05) and “**” (*P* < 0.001) indicate a significant difference from controls. “ϕ” (*P* < 0.05) and “ϕϕ” (*P* < 0.001) indicate a significant difference
