# Supplementary material for: LncRNA SNHG1 contributes to sorafenib resistance by activating the Akt pathway and is positively regulated by miR-21 in hepatocellular carcinoma cells
Source: J Exp Clin Cancer Res. 2019 May 3;38:183. doi: 10.1186/s13046-019-1177-0 (PMC6499991; doi:10.1186/s13046-019-1177-0)
Supplement: Supplementary file 1 — Supplementary Materials and Methods. (PDF 149 kb) [file 13046_2019_1177_MOESM1_ESM.pdf]

Supplementary Materials and Methods

List of antibodies, main reagents and kits…………………………......................2

Cell fraction isolation………………………..3

Cell viability analysis..3

Transfection of miRNA mimics, anti-miRNA and siRNA oligonucleotides..........4

Transfection of SNHG1 smart silencer…………………………………………...4

Assessment of apoptosis *in vitro*...5

Assessment of autophagy..5

Quantitative reverse-transcription polymerase chain reaction (qRT-PCR)............5

Plasmid constructs and luciferase assays……………………………………6

Western blot analysis..7

Immunohistochemistry and *in situ* Ki-67 proliferation index…………...........8

In situ detection of apoptotic cells...9

References...9

List of antibodies, main reagents and kits used in the study

Name	Catalogue No.	Supplier
Anti-PTEN Ab	#9552	CST
Anti-LC3A/B Ab	#12741	CST
Anti-mTOR Ab	#2972	CST
Anti-AKT Ab	#4691	CST
Anti-P-AKT (Ser473) Ab	#4060	CST
Anti-caspase-3 Ab	#9662	CST
Anti-caspase-3 Ab	#9665	CST
Anti-4EBP1 Ab	#9452	CST
Anti-p-4EBP1 Ab	#9455	CST
Anti- S6K Ab	#2708	CST
Anti-p-S6K Ab	#9208	CST
Anti-cyclin D1 Ab	#2922	CST
Anti-GSK-3β Ab	#9315	CST
Anti-P-GSK-3β Ab	#5558	CST
Anti-caspase-9 Ab	#9508	CST
GAPDH	#2118	CST
LaminA/C	#2032	CST
Anti-β-actin Ab	sc-130065	Santa Cruz
Anti-caspase-9 Ab	sc-56073	Santa Cruz
FITC-conjugated rat anti-mouse Ab	sc-516140	Santa Cruz
Anti-SLC3A2 Ab	A01794-1	BosterBio
Anti-Beclin-1	ab207612	Abcam
Mouse anti-Digoxigenin Ab	Ab420	Abcam
Cy3-conjugated goat anti-mouse Ab	ab97035	Abcam
HRP-conjugated goat anti-mouse	ab6789	Abcam
Anti-Ki67 Ab	ab15580	Abcam
HRP goat anti-mouse Ab	TA130004	OriGene
HRP goat anti-rabbit Ab	TA140003	OriGene
HRP rabbit anti-goat Ab	TA130032	OriGene
HRP rabbit anti-rat Ab	TA130038	OriGene
VECTASTAIN® ELITE ABC kit	SK-4100	Vector
TUNEL kit	# 11684795910	Sigma-Aldrich
Lipofectamine2000	#11668019	Invitrogen
CCK-8	CK04-05	Dojindo
DAPI	d9564	Sigma-Aldrich

Notes: Ab, antibody; BosterBio, Boster Biological Technology (Pleasanton, CA, USA); CST, Cell Signaling Technology (Boston, MA, USA); Santa Cruz; Santa Cruz Biotechnology (Santa Cruz, CA, USA); Cytoskeleton (Denver, CO, USA); R&D Systems (Minneapolis, MN, USA);

Sigma-Aldrich (St. Louis, MO, USA); Invitrogen (Carlsbad, CA, USA), FITC, fluorescein isothiocyanate; HRP, horseradish peroxidase; OriGene (OriGene Technologies, Inc., Beijing, China); S6K (ribosomal protein S6 kinase); TUNEL, Terminal deoxynucleotidyl transferase-mediated dUTP nick end labeling agent; Dojindo, Dojindo Molecular Technologies, Gaithersburg, MD, USA; Vector, Vector Laboratories (CA, USA); DAPI, 4',6-diamidino-2-phenylindole, GSK-3β, glycogen synthase kinase 3β. GAPDH, glyceraldehyde 3-phosphate dehydrogenase; mTOR, mammalian target of rapamycin; PTEN, phosphatase and tensin homolog; 4EBP1, eukaryotic translation initiation factor 4E-binding protein 1; LC3, microtubule-associated protein 1 light chain 3.

Cell viability assay

The Cell Counting Kit-8 (CCK-8) (Dojindo Molecular Technologies, Inc. Beijing, China) was used to determine cell viability. Cells were seeded at 1×10^3 cells /well in 96-well plates. At different time points, the culture medium was replaced with 100 μl of fresh medium containing 10μl of CCK-8 solution. The cells were further incubated for 2 h at 37°C, and the optical density (OD) at 450 nm was measured. The experiment was repeated thrice.

Cell fraction isolation

The methods have been described previously [1]. Briefly, cells were rinsed with cold DEPC-treated PBS twice, then were scraped down and centrifuged to pellet the cells. Lysis buffer was added to the pellet and pipetted up and down ten times followed by being immediately centrifuged at 8,000 rpm for 1min. The supernatant was transferred to a new tube as the cytoplasm extracts, and the remainder were considered as nuclear

fraction after washing once with wash buffer. The isolation was validated by Western blot analysis using antibody against GAPDH for cytoplasm and antibody against laminA/C for nuclear extracts.

Transfection of miRNA mimics, anti-miRNA and siRNA oligonucleotides

The methods have been described previously [2]. A siRNA targeting human SLC3A2 (SLC3A2-si) (5'-UUGCAGUGAACAGACAUCCCG-3'), a siRNA targeting SNHG1 (SNHG1-si) (5′-CAGCAGTTGAGGGTTTGCTGTGTAT-3′), miR-21 mimics (5'-AACAUCAGUCUGAUAAGCUAUU-3'), anti-miR-21 (5'-UCAACAUCAGUC UGAUAAGCUA-3') and the negative control oligonucleotides (5'-CAGUACUUUUGUGUAGUACAA-3') were purchased from GenePharma (Shanghai, China). Cells were grown to 60-70% confluence, and incubated with siRNAs at a final concentration of 0.1 μM by using Lipofectamine^{TM} 2000 (Invitrogen, Beijing, China) in a serum-free medium for 48 h and then subjected to the assays.

Transfection of SNHG1 smart silencer

RiboTM lncRNA Smart Silencer and the protocol were provided by Guangzhou RiboBio Co., Ltd., Guangzhou, China. Briefly, cells were grown to 30-50% confluence and transfected with oligonucleotides mixed with Lipofectamine 2000 at a concentration of 100 nM. The smarter silencer was a pool containing three siRNAs and three antisense oligonucleotides that target different sites of SNHG1 gene as follows: si-SNHG1 sequence 1: 5'-UGUAAGAGCAUAAACCAUGUA-3' and 5'-

CAUGGUUUAUGCUCUUACAGA-3’; si-SNHG1 sequence 2: 5’-UUACAAGA ACUGUAGUACCGG-3’ and 5’- GGUACUACAGUUCUUGUAAGG-3’; si-SNHG1 sequence 3: 5’-ACACUUUAAGGUACAUCUGAA-3’ and 5’-CAGAUGUACCUUA AAGUGUUA-3’. Antisense oligonucleotides target sequence as following: 5’-AAGACCACGAAGCCACTTACCACGA-3’, 5’-TGGCCGTAAGAACTTACTG CAACAA -3’ and 5’- ACACCAATTTGGATAATTACCTGTA-3’.

Assessment of apoptosis *in vitro*

Cells (1×10^5) were incubated in 110 μl of binding buffer containing 5μl of Annexin V and 5 μl of PI for 15 min at room temperature in dark, and then subjected to flow cytometry to measure the apoptosis rate (%) with the cytometer. Cells were also visualized under laser scanning confocal microscopy.

Autophagy assays

Cells were incubated with acridine orange (5μM) at 37°C for 15 min, washed with cold PBS, and examined by fluorescent microscopy. AVOs appeared as orange/red fluorescent cytoplasmic vesicles, while nuclei were stained green. Acridine orange-stained cells were further trypsinized and analyzed on a FACScalibur flow cytometer (BD. Biosciences, San Jose, California, USA). The degree of autophagic lysosome was expressed as fold change of acridine orange fluorescence intensity (FL3) of red in treated cells versus control cells. Tumor tissues were fixed in 2.5% glutaraldehyde solution for 1 h, washed twice with PBS, followed by further fixation with 1% Osmic

acid for 1 h, dehydrated with a graded series of ethanol, embedded, and sectioned. Sections were stained with uranium acetate and lead citrate, and observed under a transmission electron microscope (JEN-M1220, Toshiba, Japan) [13].

Quantitative reverse-transcription polymerase chain reaction (qRT-PCR)

Methods have been described in details previously [3, 4]. Briefly, total RNA was extracted from cell lysates or cytoplasm and nuclear extracts using TRIzol reagent (Invitrogen, Carlsbad, Calif., USA) according to the manufacturer's protocols. RNA quantity and quality were measured using a NanoDrop ND-1000, and RNA integrity. RNA was reverse transcribed with oligo (dT) primer into cDNA with High Efficient Reverse Transcription Kit (Toyobo). The reaction mixtures for qRT-PCR were prepared with the primers as shown in Table S1. The PCR products were analyzed by MX3000P Real-time PCR systems (Stratagen, USA). Experiments were performed in triplicate, and data were calculated by ΔΔCt methods.

Plasmid constructs and luciferase assays

Two miR-21 targeting sites in the enhancer/promoter regions of human lncRNA SNHG1 gene were established by using RNAhybrid 2.2, a tool for finding minimum free energy hybridization (http://bibiserv.techfak.uni-bielefeld.de/rnahybrid) [5] (As shown in Fig. 5A). The SNHG1 fragment containing the two putative miR-21 targeting sites, was amplified by PCR using a pair of primers, 5'-GGGCCGGCCTCTTACAGAGGAGACCTTG-3' and 5'-GGAAGCTTGTAAGA TAGAGGCAGACTG-3', and cloned into the NaeI and HindIII sites of pMIR-

REPORT vector (Ambion). This constructed reporter was named wild-type-SNHG1 (WT). To construct the mutant reporter plasmid, we used the Generate site-directed mutagenesis system (Invitrogen) to introduce mutations into the putative miR-21 targeting sites of the wild-type-SNHG1 vector. Three mutant reporters were constructed (Mut1, “TAAGCT” [62622089-62622094 nt] mutated to “ATTCGA”; Mut2, “TAAGCT” [62622157-62622162 nt] mutated to “ATTCGA”; Mut3, the combination of Mut1 and Mut 2) (Fig. 5). Luciferase reporter transfection and dual-luciferase assays were performed as described previously [3]. Briefly, the reporter vector plasmid was transfected into cells using Lipofectamine 2000. To correct transfection efficiency, an empty luciferase reporter vector without the miR-21 target was transfected in parallel. Luciferase activities in cells were measured by using a luciferase assay kit (Promega, Madison, WI) and were expressed as ratios of the luciferase activity of the reporter vector with miR-21 targeting sequence over the one without the miR-21 targeting sequence.

Western blot analysis

The methods have been described previously [6, 7]. Cells or tumor tissues were homogenized in protein lysate buffer (50 mM Tris pH 7.4, 100 μM EDTA, 0.25 M sucrose, 1%SDS, 1% NP40, 1μg/ml leupeptin, 1μg/ml pepstatin A and 100 μM phenyl methyl sulfonyl flouride) and debris was removed by centrifugation at 10,000 × g for 10 min at 4°C. Protein concentrations of cell or tissue lysates were determined using the Bio-Rad protein assay (Bio-Rad, Richmond, CA, USA). Lysates were resolved on

sodium dodecyl sulfate-polyacrylamide (SDS-PAGE) gels, electrophoretically transferred to polyvinylidene difluoride (PVDF) membranes. The membranes were blocked in TBST (137 mM NaCl, 20 mM Tris HCl [pH 7.6], and 0.1% [v/v] Tween 20) containing 5% (w/v) nonfat dry milk at 37°C for 2 h, and then incubated overnight with primary Abs, and subsequently with alkaline phosphatase-conjugated secondary Abs for 2 h at room temperature in the dark. They were developed with 5-bromo-4-chloro-3-indolyl phosphate (BCIP)/ nitro blue tetrazolium (NBT) (Tiangen Biotech Co. Ltd., Beijing, China). The density of each band was measured using a densitometric analysis program (FR200, Shanghai, China). In preliminary experiments, serial dilutions of lysates (containing 2.5, 5, 10, 20, 40 or 80μg protein) were immunoblotted; band intensities were measured and plotted against protein amounts to generate a standard curve, and the amount of protein for each blot was determined.

Immunohistochemistry and *in situ* Ki-67 proliferation index

Formalin fixed tumor specimens were transferred to 70% ethanol, and subsequently paraffin-embedded and sectioned. Tumor sections were rinsed with PBS, blocked with 3% BSA for 2 h, and incubated with anti-PEA-15 or anti-Ki-67 Abs at 4°C overnight. They were subsequently incubated for 30 min with the appropriate secondary Ab using the Ultra-Sensitive TMS-P kit (Zhongshan Co., Beijing, China), and immunoreactivity developed with Sigma FAST DAB (3,3’-diaminobenzidine tetrahydrochloride) and $CoCl_2$ enhancer tablets (Sigma-Aldrich, Shanghai, China). Sections were counterstained with hematoxylin, mounted, and examined by microscopy. Tumoral

expression of PEA-15 was examined under microscopy. The Ki-67 positive cells were counted in 10 randomly selected × 400 high-power fields under microscopy. The Ki-67 proliferation index was calculated according to the following formula: the number of Ki-67 positive cells/ the total cell count × 100%.

In situ detection of apoptotic cells

Tumor cryosections were prepared 4 days after intratumoral injection of shRNAs, and stained with the TUNEL (Terminal deoxynucleotidyl transferase dUTP nick end labeling) (Roche, Shanghai, China). The TUNEL positive cells were counted in 20 randomly selected × 200 high-power fields under microscopy. The apoptosis index was calculated according to the following formula: the number of apoptotic cells × /total number of nucleated cells × 100%.

References

[1] P. Wang, J. Xu, Y. Wang, X. Cao, An interferon-independent lncRNA promotes viral replication by modulating cellular metabolism, Science (New York, N.Y.) 358(6366) (2017) 1051-1055.

[2] W. Jiang, G. Li, W. Li, P. Wang, P. Xiu, X. Jiang, B. Liu, X. Sun, H. Jiang, Sodium orthovanadate overcomes sorafenib resistance of hepatocellular carcinoma cells by inhibiting Na(+)/K(+)-ATPase activity and hypoxia-inducible pathways, Scientific reports 8(1) (2018) 9706.

[3] C. He, X. Dong, B. Zhai, X. Jiang, D. Dong, B. Li, H. Jiang, S. Xu, X. Sun, MiR-

21 mediates sorafenib resistance of hepatocellular carcinoma cells by inhibiting autophagy via the PTEN/Akt pathway, Oncotarget 6(30) (2015) 28867-81.

[4] B. Zhai, F. Hu, X. Jiang, J. Xu, D. Zhao, B. Liu, S. Pan, X. Dong, G. Tan, Z. Wei, H. Qiao, H. Jiang, X. Sun, Inhibition of Akt reverses the acquired resistance to sorafenib by switching protective autophagy to autophagic cell death in hepatocellular carcinoma, Molecular cancer therapeutics 13(6) (2014) 1589-98.

[5] M. Rehmsmeier, P. Steffen, M. Hochsmann, R. Giegerich, Fast and effective prediction of microRNA/target duplexes, RNA (New York, N.Y.) 10(10) (2004) 1507-17.

[6] L. Li, X. Jiang, Q. Zhang, X. Dong, Y. Gao, Y. He, H. Qiao, F. Xie, X. Xie, X. Sun, Neuropilin-1 is associated with clinicopathology of gastric cancer and contributes to cell proliferation and migration as multifunctional co-receptors, Journal of experimental & clinical cancer research : CR 35(1) (2016) 16.

[7] Z. Wei, X. Jiang, H. Qiao, B. Zhai, L. Zhang, Q. Zhang, Y. Wu, H. Jiang, X. Sun, STAT3 interacts with Skp2/p27/p21 pathway to regulate the motility and invasion of gastric cancer cells, Cellular signalling 25(4) (2013) 931-8.
