# Supplementary material for: LncRNA SNHG1 contributes to sorafenib resistance by activating the Akt pathway and is positively regulated by miR-21 in hepatocellular carcinoma cells
Source: J Exp Clin Cancer Res. 2019 May 3;38:183. doi: 10.1186/s13046-019-1177-0 (PMC6499991; doi:10.1186/s13046-019-1177-0)
Supplement: Supplementary file 2 — Supplementary tables and figures. (PDF 149 kb) [file 13046_2019_1177_MOESM2_ESM.pdf]

Supplementary Tables

Table S1. Ten potential lncRNAs predicted to have binding sites with has-miR-21-5p

<table>
<tr><th>lncRNA</th><th>miRNA</th><th>Interaction</th><th>lncRNA chr</th><th>Binding Start</th><th>Binding End</th><th>Energy (kCal/Mol)</th><th>Score</th></tr>
<tr><td>lnc-ANG-1:1</td><td>hsa-miR-21-5</td><td>Q: 3' agUUGUAGUCAGACUAUUCGAu 5’
::| |: | | |||||||
R: 5' tgGGCTTTTCTGTAATAAGCTt 3'</td><td>chr14</td><td>21,168,168</td><td>21,168,189</td><td>-10.54</td><td>148</td></tr>
<tr><td>lnc-ATL3-1:1</td><td>hsa-miR-21-5p</td><td>Q: 3' aguUGUAGUCAGACUAUUCGAu 5'
::||||| ||||||||
R: 5' cgtGTATCAG--AGATAAGCTg 3'</td><td>chr11</td><td>63,529,696</td><td>63,529,677</td><td>-18.63</td><td>156</td></tr>
<tr><td>lnc-HOXC4-3:1</td><td>hsa-miR-21-5p</td><td>Q: 3' aguuGUAGUCA-GACUAUUCGAu 5'
|| | | | |||||||
R: 5' cttcCAGCCCTGCCCATAAGCTa 3'</td><td>chr12</td><td>54,526,176</td><td>54,526,198</td><td>-14.16</td><td>141</td></tr>
<tr><td>lnc-LINC00273-5:1</td><td>hsa-miR-21-5p</td><td>Q: 3' aguuGUAGUCAGACUAUUCGAu 5'
||||| | |||||||
R: 5' cattCATCA-TAATATAAGCTt 3'</td><td>chr16</td><td>34,428,887</td><td>34,428,867</td><td>-12.26</td><td>152</td></tr>
<tr><td>lnc-LRRD1-1:1</td><td>hsa-miR-21-5p</td><td>Q: 3' agUUGUAGUCAGACUAUUCGAu 5'
||:||| || |||||||
R: 5' atAATATC---CTCATAAGCTa 3'</td><td>chr7</td><td>91,829,567</td><td>91,829,549</td><td>-15.41</td><td>156</td></tr>
<tr><td>lnc-TUG1-2:7</td><td>hsa-miR-21-5p</td><td>Q: 3' aguUGUAGUCAGACUAUUCGAu 5'
| || || || |||||||
R: 5' gatAAATGAG-CTAATAAGCTt 3'</td><td>chr22</td><td>31,372,028</td><td>31,372,048</td><td>-11.33</td><td>157</td></tr>
<tr><td>lnc-RP11-293M10.1.1-1:1</td><td>hsa-miR-21-5p</td><td>Q: 3' aguUGUAGUCAG-ACUAUUCGAu 5'
::| :|||| ||||||||
R: 5' tatGTAATAGTCAGGATAAGCTa 3'</td><td>chr14</td><td>75,891,465</td><td>75,891,443</td><td>-18.89</td><td>158</td></tr>
<tr><td rowspan="2">lnc-SNHG1-1:11</td><td>hsa-miR-21-5p</td><td>Q: 3' aguuGUAGUCAG-AC-UAUUCGAu 5'
::|:: |: || |||||||
R: 5' cagtTGTTGTTTATGAATAAGCTt 3'</td><td>chr11</td><td>62,622,094</td><td>62,622,071</td><td>-10.02</td><td>144</td></tr>
<tr><td>hsa-miR-21-5p</td><td>Q: 3' agUUGUAGUCAGACUAUUCGAu 5'
:|: | | | |||||||||
R: 5' atGATTTGAAT-TGATAAGCTg 3'</td><td>chr11</td><td>62,622,162</td><td>62,622,142</td><td>-14.7</td><td>154</td></tr>
<tr><td>lnc-ZNF131-1:5</td><td>hsa-miR-21-5p</td><td>Q: 3' aguUGUAGUCAGACUAUUCGAu 5'
::||: || |||||||
R: 5' aacGTATTTTTCCCATAAGCTt 3'</td><td>chr5</td><td>43,045,093</td><td>43,045,114</td><td>-11</td><td>151</td></tr>
<tr><td>lnc-GAS5-1:2</td><td>hsa-miR-21-5p</td><td>Q: 3' agUUGUAGUCAGAC---UAUUCGAu 5'
||:||| :|| |||||||
R: 5' taAATATCCTGTTGCAAATAAGCTt 3'</td><td>chr5</td><td>69,282,791</td><td>69,282,815</td><td>-13.51</td><td>151</td></tr>
</table>

Explanations and abbreviations: lnc-ANG, Angiogenin; lnc-ATL3, Atlastin GTPase 3; lnc-HOXC4, Homeobox C4; lnc-LINC00273, Long intergenic non-protein coding RNA 273; lnc-LRRD1, Leucine rich repeats and death domain containing 1; lnc-TUG1, Taurine-upregulated gene 1; lnc-RP11-293M10.1.1, Ergosterol biosynthetic protein28; lnc-SNHG1, Small nucleolar RNA host gene 1; lnc-ZNF131, Zinc finger protein 131; lnc-GAS5-1, Growth arrest specific 5.

Table S2. Primers used for qRT-PCR in the study

Genes	Forward (5'→3')	Reverse (5'→3')
lnc-ANG-1:1	CCCCACCTAGATGCAAAGCA	AAACGGGCAGGACAATGGAA
lnc-ATL3-1:1	AAGGGCTGCGGTAGTGATTC	CTAGGCAAGAGGGCCAACTC
lnc-HOXC4-3:1	GCTTCCGTCTTTGGGTTCTT	TGAGGCTGATGGAGGCATG
lnc-LINC00273-5:1	CTATGTCATCCTCGGTGGGC	CGAGTGATTGAAGGCAGGGT
lnc-LRRD1-1:1	TGAGCATTAGCGGTGTCTGAG	GAAAAGCCTACTACAGCCTTCATTA
lnc-TUG1-2:7	TCCTTGTTTAGTGCATCTTTGCC	TGAGTGGTTATTCTGATAGCCTGC
lnc-RP11-293M10.1.1-1:1	CACAAACTGAGAGCGACAGG	AATGCCTTCATCTCAGCCCAT
lnc-SNHG1-1:11	GTGTGCTGTTACCCTCATCCA	ACAACAACTGCCATCAGAACTCTA
lnc-ZNF131-1:5	ACGCTCGTTGATTCTGATGG	TCTTGTGCTGGAGTCTTGCAG
lnc-GAS5-1:2	GTGAGGTATGGTGCTGGGTG	GCTTTCTGTCTAATGCCTGTGTG
microRNA-21	CCCGCCTAGCTTATCAGACTG	GCCGTCGGTGTCAACATCA
PTEN	CAAGATGATGTTTGAAACTATTCCAATG	CCTTTAGCTGGCAGACCACAA
U6	CTCGCTTCGGCAGCACA	AACGCTTCACGAATTTGCGT
U1	GAAACTCGACTGCATAATTTGTGGTAG	CTTGGCGTACAGTCTGTTTTTGAAACTC
SLC3A2	TGTAAAACGACGGCCAGT	CAGGAAACAGCTATGACC
GAPDH	CACCCATGGCAAATTCCATGGCA	TCTAGACGGCAGGTCAGGTCCACC

Explanations and abbreviations: lnc-ANG, Angiogenin; lnc-ATL3, Atlastin GTPase 3; lnc-HOXC4, Homeobox C4; lnc-LINC00273, Long intergenic non-protein coding RNA 273; lnc-LRRD1, Leucine rich repeats and death domain containing 1; lnc-TUG1, Taurine-upregulated gene 1; lnc-RP11-293M10.1.1, Ergosterol biosynthetic protein28; lnc-SNHG1, Small nucleolar RNA host gene 1; lnc-ZNF131, Zinc finger protein 131; lnc-GAS5-1, Growth arrest specific 5; PTEN, phosphatase and tensin homolog; SLC3A2, glyceraldehyde 3-phosphate dehydrogenase.

Supplementary Figures

Figure S1.

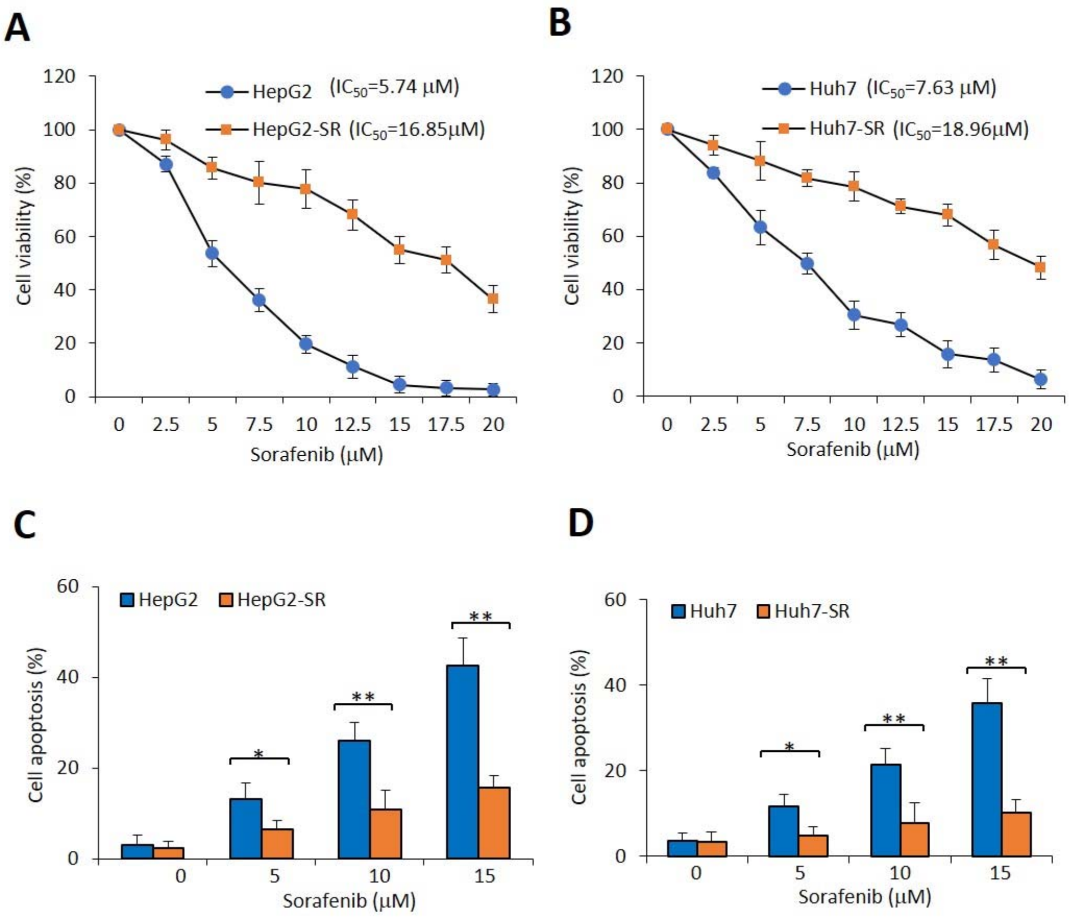

Figure S1. Sorafenib-resistant HCC cells are more refractory to sorafenib treatment than parental HCC cells. (A, B) Sorafenib-resistant cells (HepG2-SR and Huh7-SR) and their parental cells (HepG2 and Huh7) were incubated for 48 h with sorafenib at various concentrations as indicated. Cell viability (%) was compared with the respective untreated cells. The values of IC_{50} for each cell type were calculated. (C, D) The above cells were incubated for 48 h with 0, 5, 10 or 15 μM of sorafenib, and then analyzed to measure apoptosis rate (%). "*" indicates $P<0.05$, and "**", $P<0.001$.

Figure S2

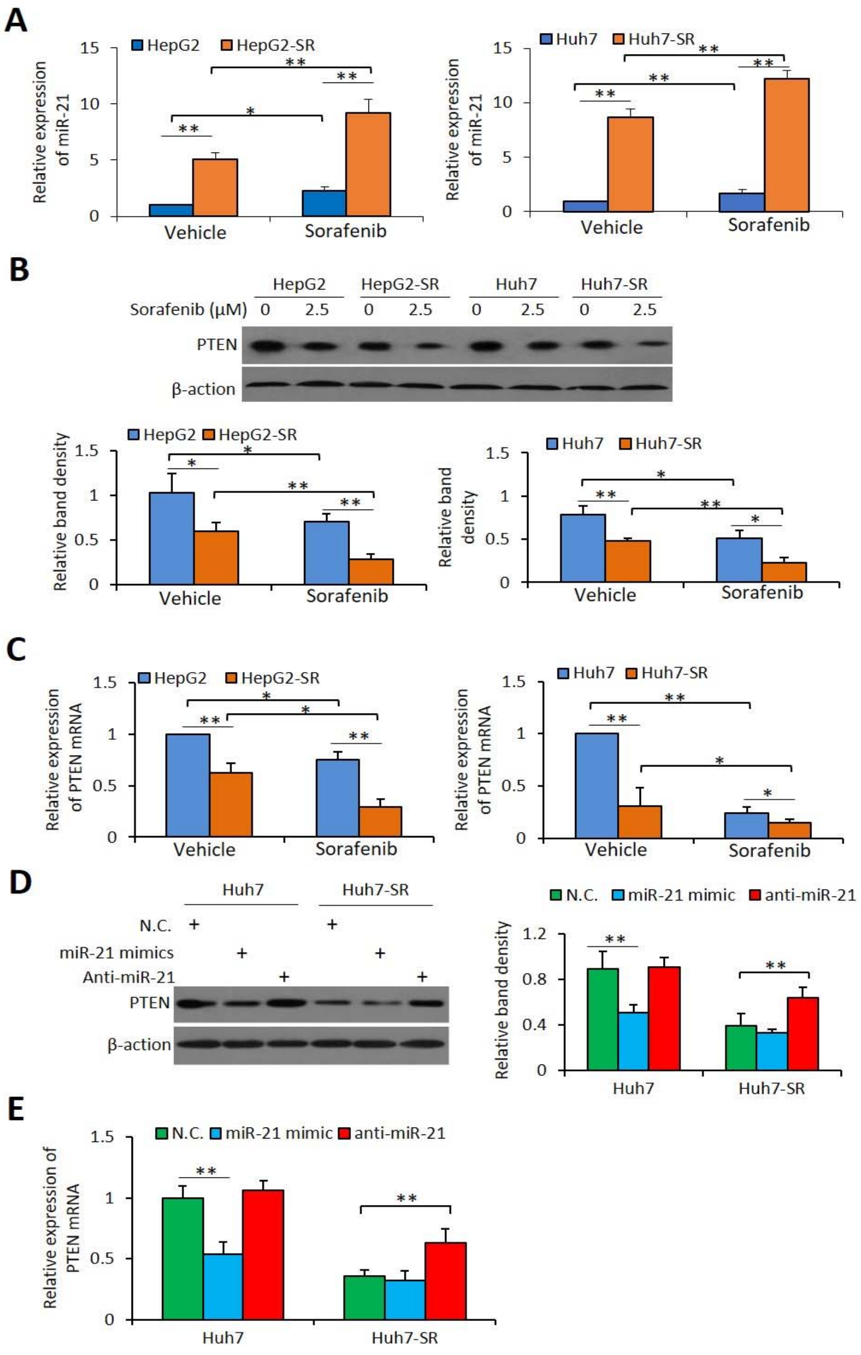

Figure S2. Exposure to sorafenib induces downregulation of PTEN by upregulating miR-21. (A-C) HepG2, HepG2-SR, Huh7 and Huh7-SR cells were incubated with sorafenib (2.5 μM) for 48 h. (A) The expression of miR-21 in the above cells was measured by qRT-PCR, and the level of miR-21 from untreated parental cells was defined as 1. (B, C) The above cells were immunoblotted by using an anti-PTEN Ab (B) and were subjected to qRT-PCR to measure PTEN mRNA (C). (D, E) Huh7 and Huh7-SR cells were transfected with negative control oligonucleotides (N.C.), miR-21 mimics or anti-miR-21 for 24 h. The above cells were subjected to immunoblotting (D) or to qRT-PCR to measure PTEN mRNA (E). The density of each band was normalized to respective β-actin. The relative expression level in untreated parental cells was defined as 1.0. "*" ($P<0.05$) and "**" ($P<0.001$) indicate a significant difference.

Figure S3

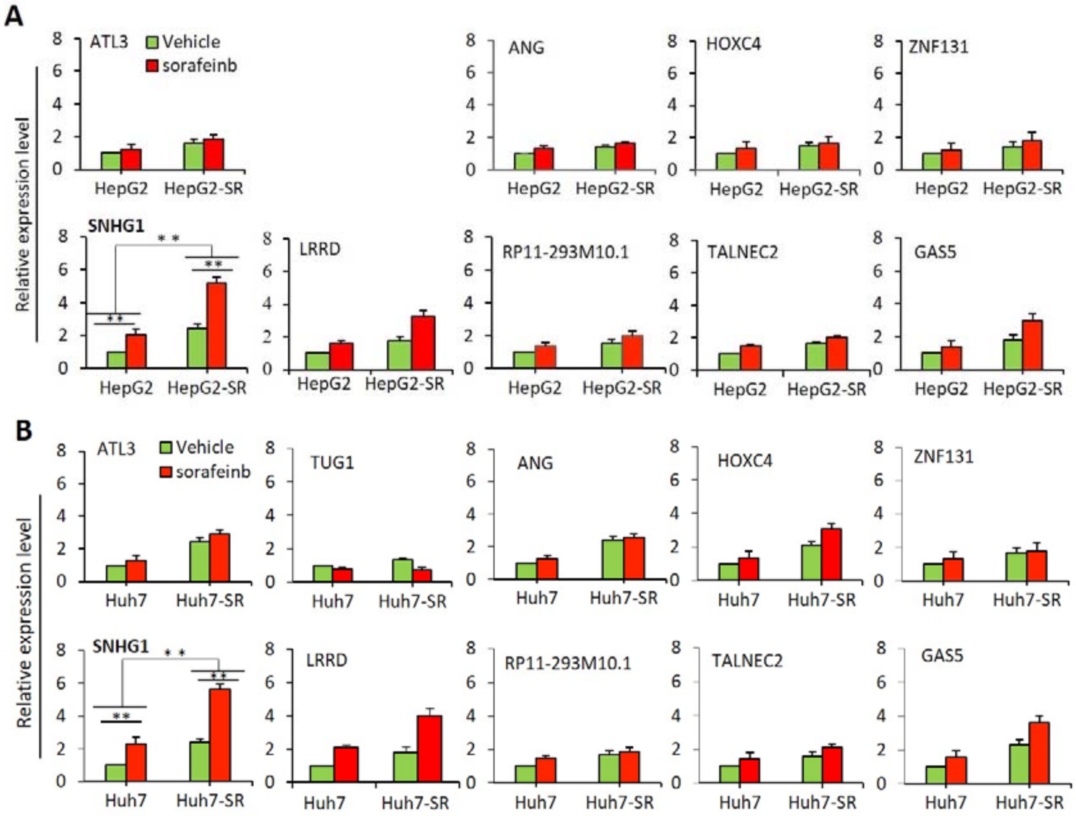

Figure S3. SR-HCC cells overexpress SNHG1 and sorafenib incubation elevates the expression of SNHG1 in HCC cells. The expression levels of ten potential lncRNAs as indicated were examined by qRT-PCR in Huh7/Huh7-SR (A) and HepG2/HepG2-SR (B) cells, which were incubated with vehicle or sorafenib (5μM) for 48 h. "*" (P<0.05) and "**" (P<0.001) indicate a significant difference.

Figure S4

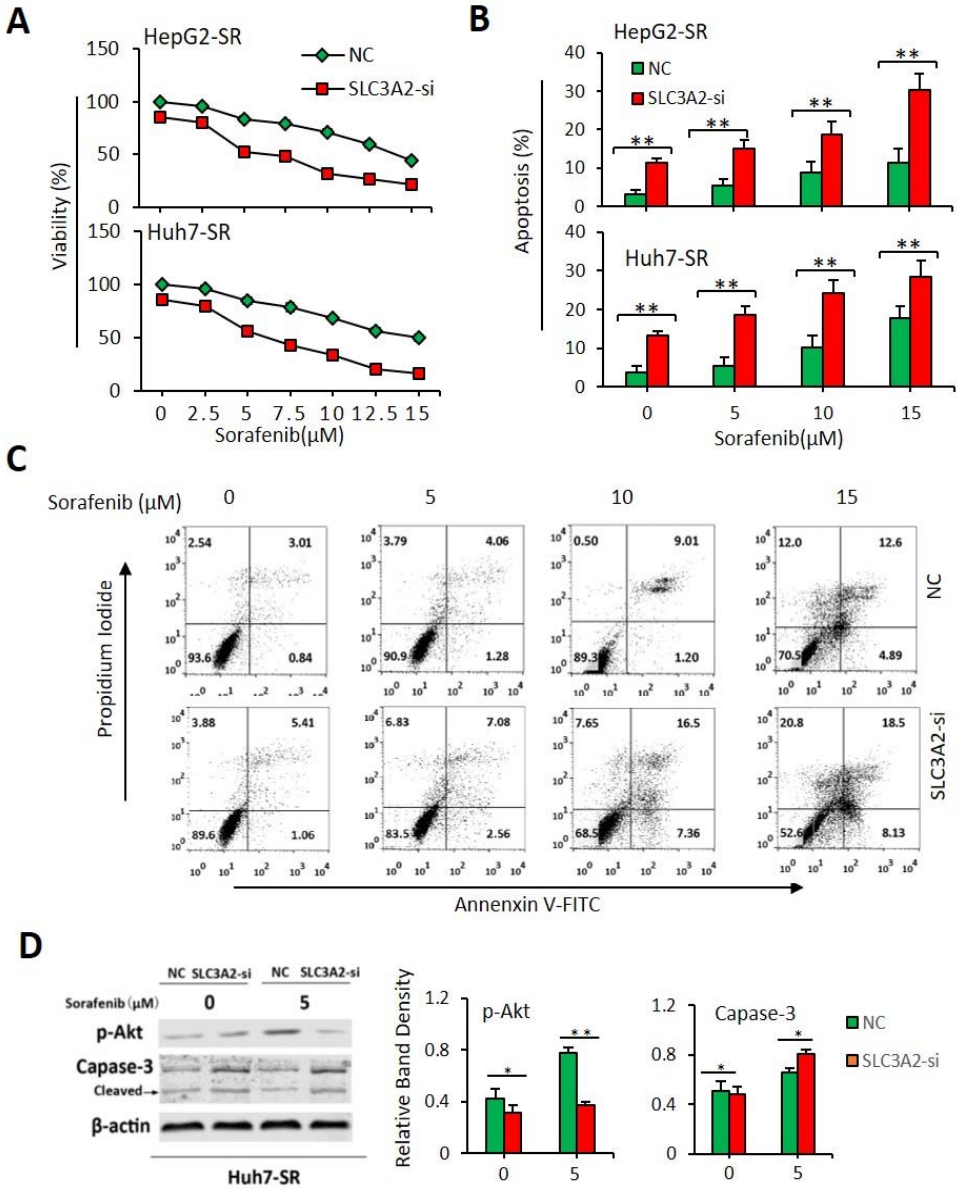

Figure S4. Depletion of SLC3A2 enhances the activity of sorafenib in suppressing sorafenib-resistant HCC cells. (A) HepG2-SR and Huh7-SR cells were transfected with negative control (NC) and siRNA targeting SLC3A2 (SLC3A2-is) were incubated for 48 h in culture media containing serial concentrations of sorafenib (2.5, 5, 7.5, 10, 12.5 and 15μM). Cell viability was measured by CCK-8 and normalized to cells incubated with medium only. (B, C) HepG2-SR and Huh7-SR cells transfected with negative control (NC) and siRNA targeting SLC3A2 (SLC3A2-is) were incubated for 48 h in culture media containing various concentrations of sorafenib (0, 5, 10 and 15μM). (B) The above cells were subjected to cytometry for analyzing apoptosis. (C) Representative dot plots were from the above cytometrically analyzed Huh7-SR cells, which were also subjected to Western blot analysis (D). The density of each band was normalized to β-actin. "*" ($P<0.05$) and "**" ($P<0.001$) indicate a significant difference.

Figure S5

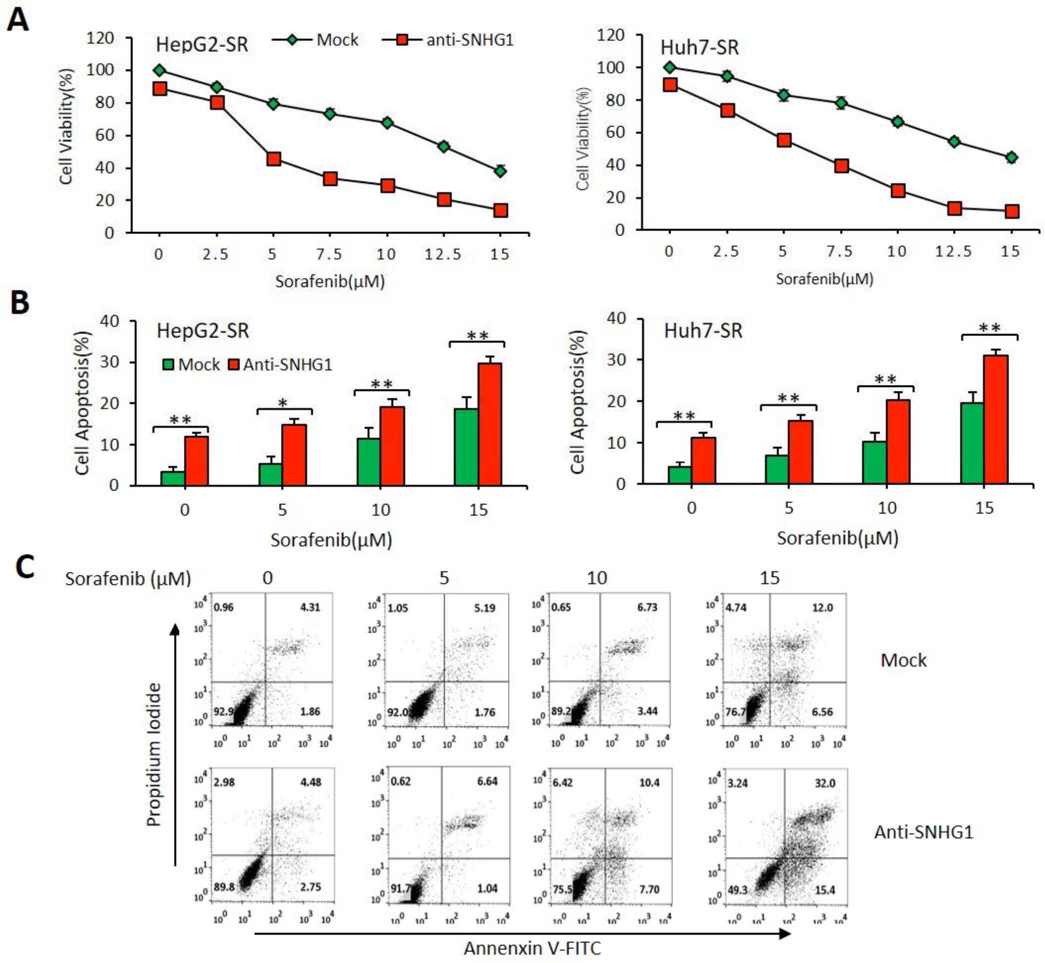

Figure S5. Depletion of SNHG1 inhibits the proliferation and induces the apoptosis of sorafenib-resistant HCC cells. HepG2-SR and Huh7-SR cells were either untreated (Mock) or transfected with SNHG1-smart Silencer (anti-SNHG1) were incubated for 48 hours with sorafenib at various concentrations as indicated. (A) Cell viability (%) was compared with the respective untreated cells. (B) The above cells incubated with 0, 5, 10 or 15μM of sorafenib were analyzed to measure apoptosis rate (%). (C) Representative dot plots were from the above cytometrically analyzed Huh7-SR cells. "*" ($P<0.05$) and "**" ($P<0.001$) indicate a significant difference.

Figure S6

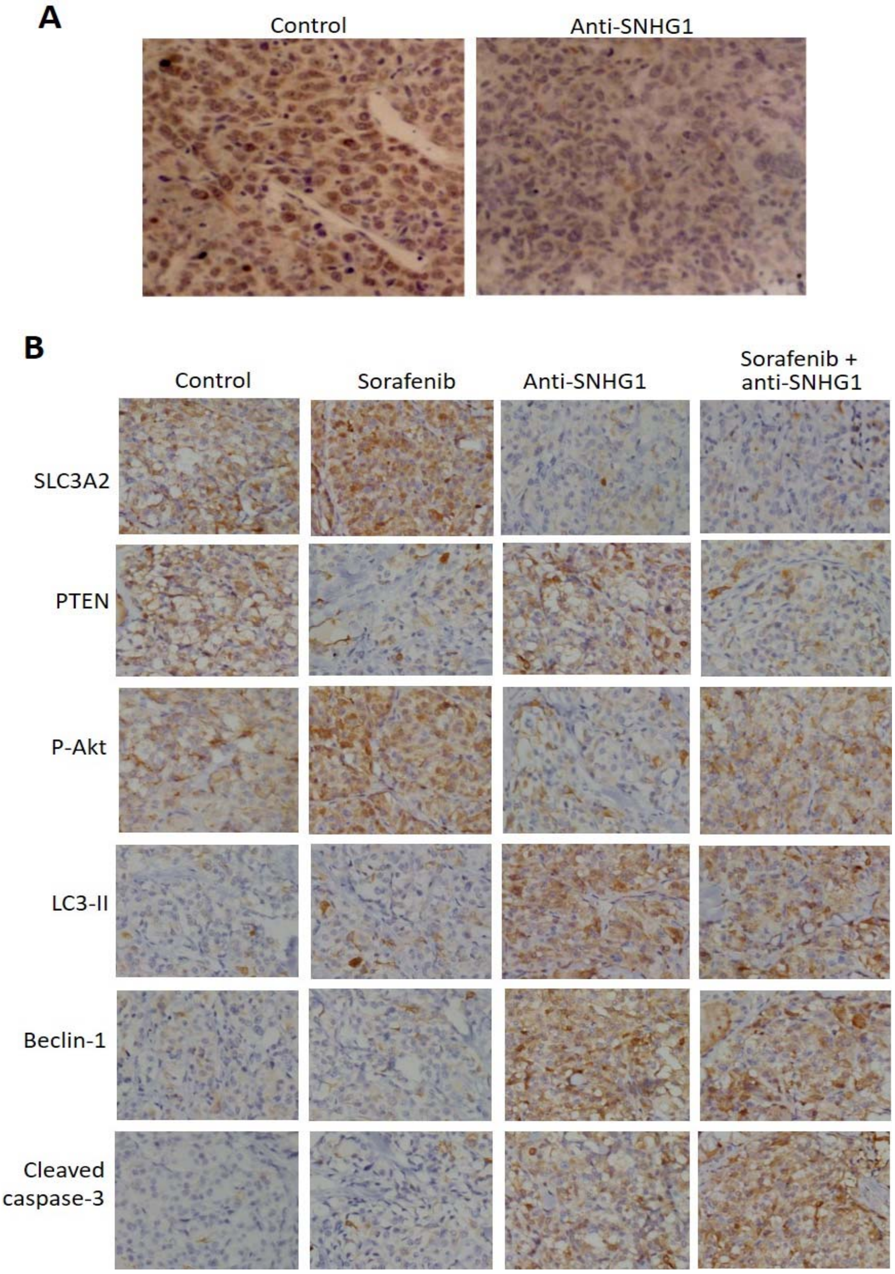

Figure S6. Gene expression *in vivo*. (A) Tumors were harvested 2 days after treatments from mice in the control and anti-SNHG1 groups in Fig. 4A. Representative images were taken from tumor sections subjected to in situ hybridization. (B) Representative images of tumor sections taken from Figure 4 were immunostained with antibodies against SLC3A2, PTEN, p-Akt, LC3-II, Beclin-1 and cleaved caspase-3.
